# Isolation, Synthesis and Absolute Configuration of the Pericharaxins A and B, Epimeric Hydroxy-Polyene Glycerol Ethers from the Calcarean Sponge *Pericharax heteroraphis*

**DOI:** 10.3390/md20100635

**Published:** 2022-10-11

**Authors:** Capucine Jourdain de Muizon, Céline Moriou, Sylvain Petek, Merrick Ekins, Marthe Rousseau, Ali Al Mourabit

**Affiliations:** 1 CNRS, Institut de Chimie des Substances Naturelles, Université Paris-Saclay, F-91190 Gif-sur-Yvette, France; 2 IRD, CNRS, Ifremer, University Brest, LEMAR, F-29280 Plouzane, France; 3 Queensland Museum, P.O. Box 3300, South Brisbane, QLD 4101, Australia; 4 School of Biological Sciences, University of Queensland, St Lucia, QLD 4072, Australia; 5 Griffith Institute for Drug Discovery, Griffith University, Brisbane, QLD 4111, Australia; 6 UMR5510 MATEIS, CNRS, INSA-Lyon, University of Lyon, F-69621 Lyon, France; 7 U1059 SAINBIOSE, INSERM, Mines Saint-Étienne, University Jean Monnet, F-42023 Saint-Étienne, France

**Keywords:** marine metabolites, calcareous sponge, *Pericharax*, glycerol ether, pericharaxin

## Abstract

Naturally occurring epimeric hydroxy-polyene glycerol ether pericharaxins A (**1a**) and B (**1b**) were isolated from the calcarean sponge *Pericharax heteroraphis*. The structural and stereochemical characterization of both diastereoisomers were established on the basis of spectroscopic data analysis and total synthesis in seven steps. The mixture of pericharaxins A (**1a**) and B (**1b**) was proven to be epimeric by chiral-phase HPLC analysis of both synthetic and natural samples. Further separation of the epimers and application of Mosher’s method to the synthetic compounds allowed unequivocal absolute configuration assignment. While natural products and the synthetic intermediates were shown to be non-cytotoxic on the HCT116 cell line, the endochondral differentiation activity using human type X collagen transcription activity in ATDC5 cells is interesting.

## 1. Introduction

In the last decade, the developments in metabolomics and molecular networks constructed by LCMS analysis on minor metabolites have often provided only minute quantities and partial structural elements of natural products. Thus, complete structural and bioactivity studies of such metabolites undoubtedly require either the isolation of the pure molecule or its synthesis in sufficient quantities. Structural misassignment of natural products remains relatively common [1]. Consequently, organic synthesis is more and more integrated in the process of natural products studies and often used, whenever possible, to support unambiguous structural characterization. Synthesis can even be anticipated in order to use the spectral data of synthesized compounds in synthesis-based reverse metabolomics [2].

While screening marine calcareous sponges for bioactive compounds, molecular networking analysis allowed discovery of several new clusters different from those belonging to the well-known major 2-aminoimidazolone alkaloids and their transition Zn metal complexes [3,4]. One of the new clusters which did not appear to contain nitrogen atoms attracted our attention (Figure S1, page S3). We thus describe herein the isolation of novel epimeric glycerol ethers **1a** and **1b** from the sponge *Pericharax heteroraphis* and their total synthesis allowing attribution of their absolute configuration.

## 2. Results and Discussion

### Isolation and Structure Elucidation

The sponge *Pericharax heteroraphis* was collected in the Wallis Island lagoon during the Wallis 2018 expedition [5]. The isolation of compounds **1a** + **1b** was achieved by the extraction of the freeze-dried sponge with a mixture of CH_2_Cl_2_/MeOH: 1/1 and further *n*-BuOH/H_2_O partitioning. Focusing on the potential new compounds, the butanolic extract was fractionated by reversed-phase C18 flash chromatography and further purified by repetitive preparative C18-HPLC. The NMR spectra of the two epimers **1a** + **1b** were extremely homogeneous and seemed to indicate a single compound. Thus, structural determination was carried out on the mixture until later discovery of the presence of the two epimers by comparison with the synthetic sample.

**Figure 1:**
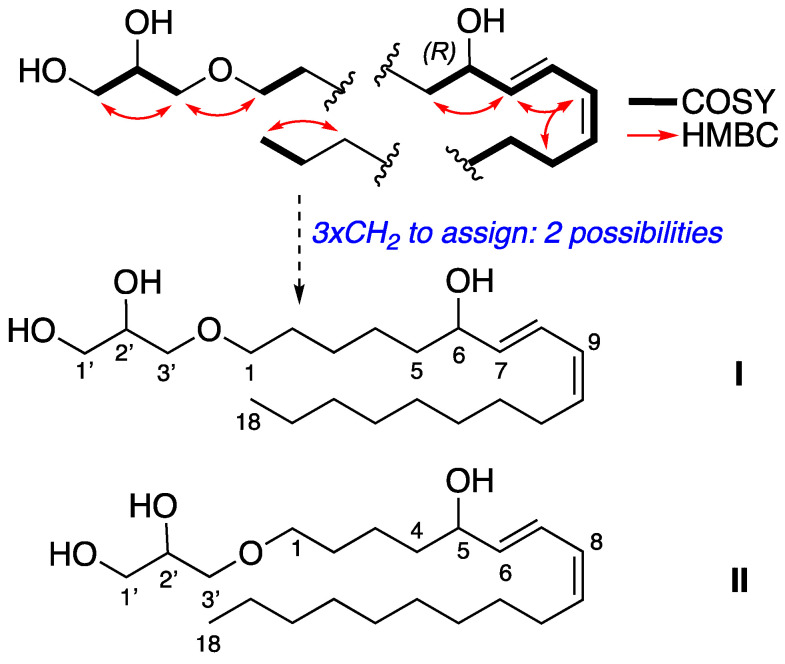
Fragments assigned by mass spectrometry and NMR spectroscopy and the resulting I/II structural proposals.

The common molecular formula of **1a** and **1b** was determined to be C_21_H_40_O_4_ by high-resolution mass spectrometry analysis (HRESIMS *m*/*z* [M + Na]^+^ 379.2799) with 2 degrees of unsaturation. ^1^H and ^13^C NMR spectra showed two pairs of olefinic methine resonances at *δ*_H_/*δ*_C_ 5.67/136.0, 6.49/125.9, 5.98/127.9, and 5.44/133.4 which were correlated to each other in the COSY spectrum as well as to a proton of one of the oxymethine groups at *δ*_H_ 4.17/73.0 (Figure 1). The corresponding dienol system accounted for the two sites of unsaturation required by the molecular formula. The secondary alcohol corresponding to *δ*_C_ 73.0 was confirmed by the loss of H_2_O (18 units) at 339.2858 [M + H − H_2_O]^+^ in the mass spectrum. The two disubstituted and conjugated *E* and *Z* geometries of the olefins were assigned to be a part of a (2*E*,4*Z*)-hexa-2,4-dien-1-ol- motif on the basis of their 15.4 and 11.0 Hz coupling constants. The glycerol partial structure was evident based on the well-resolved signals in the ^1^H and ^13^C NMR 1D and COSY spectra. The chemical shift values at *δ_H_* 3.53, 3.87, and 3.69 corresponded to the C1’–C3’ glycerol unit. The latter glycerol fragment was confirmed by the loss of 92 units at 247.2405 [M + H − H_2_O − C_3_H_8_O_3_]^+^ in the mass spectrum. At this stage, a terminal methyl and 12 methylenes remained to be assigned. Further COSY, HSQC, and HMBC data analysis converged to a glycerol ether bearing a hydroxydiene motif and two series of n and m methylenes whose sum n + m was 12 (Figure 1).

A literature survey revealed a paper describing the ceratodictyols, that is, two pairs of mixtures of allylic alcohols isolated from an alga/sponge association [6]. Because compound **1** appeared different from the compound described by these authors, unambiguous assignment of the structure needed additional studies to unequivocally assign the structure of one of the two isomers, I or II, shown in Figure 1.

During the course of manipulation, it was noticed that compound **1** was rather unstable. Its lability is probably due to the sensitivity of the hydroxydiene function. Oxidative degradation of the labile *E*,*Z*-penta-2,4-dien-1-ol moiety resulted in additional complex which, together with the small quantity available, hampered further structural studies. The resulting difficulty in discriminating between the two proposed regioisomers I and II thus encouraged us to consider their separate synthesis. This would also allow unambiguous assignment of the absolute configuration at the C-2’ stereogenic center of the glycerol moiety. Still ignoring that that the isolated product **1** was a mixture of epimers **1a** + **1b**, we targeted the synthesis of the epimeric mixtures of both regioisomers with the intention of separating and comparing them to the natural product. Initially, we assumed an (*S*)-configuration of the C-2’ carbon of glycerol as described for other marine glycerol ethers ceratodictyols [6].

Consequent synthetic studies have been previously reported [7,8]. Unfortunately, synthesis of hydroxydiene units is rather rare. Preliminary tentative synthesis of the conjugated hydroxydiene inspired by the work of Schmidt and coworkers was not successful [9].

**Scheme 1:**
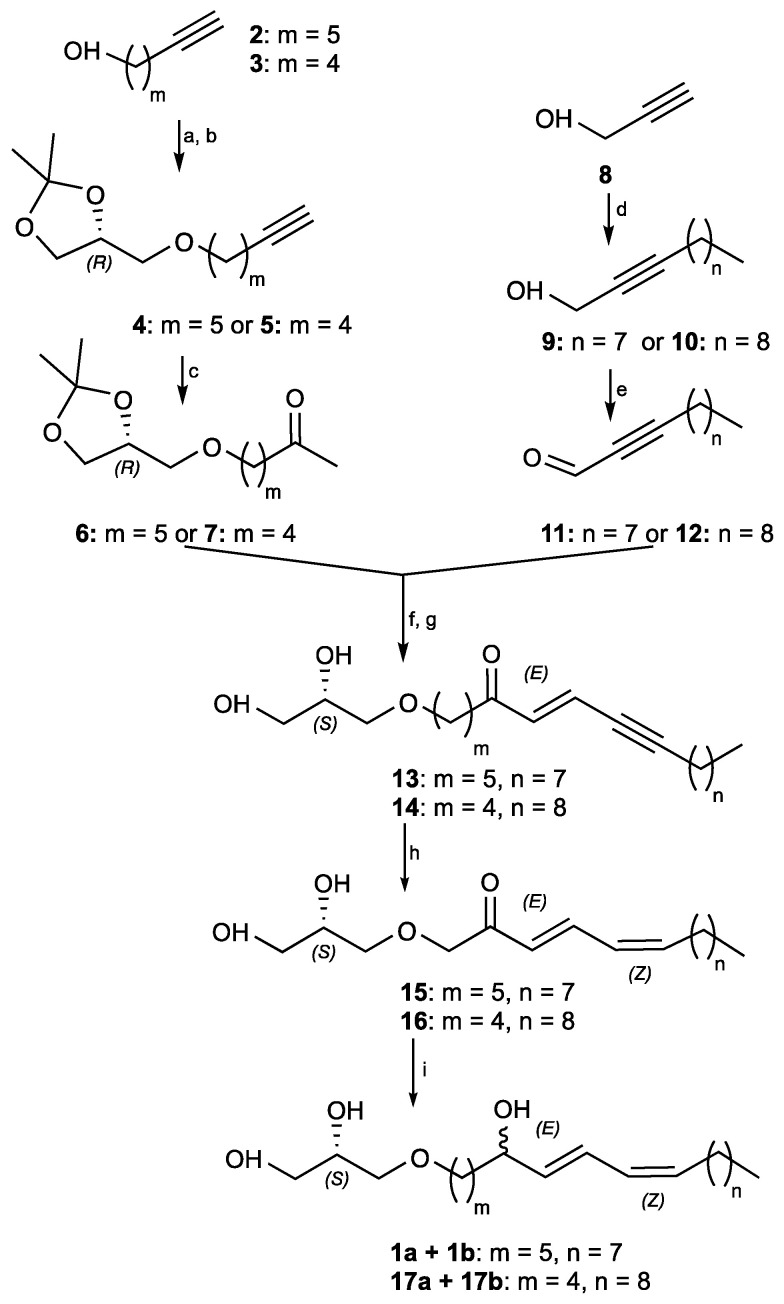
Synthesis of glycerol ethers **1** and **17**. Reagents and conditions for m = 5 and n = 7: (a) MsCl, triethylamine in THF, 2 h, from 0 °C to r.t., quant.; (b) (*R*)-4-hydroxymethyl-2,2-dimethyl-1,3-dioxolane, NaH 90% in DMF, overnight from 0 °C to r.t., 58%; (c) Hg(OAc)_2_, PPTS in THF/pH 7 buffer 9:1, 0 °C, then 3 h 30 at 50 °C, 60%; (d) BrC_n_H_2n+1_, *n*-BuLi, HMPA in THF 30 min at −78 °C then overnight at r.t., 82%; (e) DMP in CH_2_Cl_2_, 30 min at 0 °C, quant.; (f) *n*-BuLi, (i-Pr)_2_NH in THF, 5 min at −78 °C then 15 min at r.t.; (g) HCl in THF/H_2_O: 5/1, overnight at 65 °C, 47% over 2 steps; (h) quinoline, Pd Lindlar under H_2_ atmosphere, in heptane/AcOEt: 8/2, 1 h 15 at 0 °C, not purified; (i) NaBH_4_ in MeOH 30 min at 0 °C, 11% over 2 steps (overall yield = 1.5%).

Structurally, compound **1** is a linear glyceryl ether assembly of glycerol and the chain bearing the hydroxydiene unit. As shown in Scheme 1, synthesis of regioisomer A was initiated using commercially available (*R*)-4-hydroxymethyl-2,2-dimethyl-1,3-dioxolane (enantiopure l,2-O-isopropylideneglycerol) and the acetylenic derivative **2** (the homolog **3** of the latter was used for synthesis of regioisomer II using a similar synthetic strategy as for I).

The first synthetic pathway considered was through a Wittig coupling by analogy, with Andersen’s synthesis of niphatenones [10]. In our hands, the reported phosphonium salts were successfully obtained, but their key coupling reaction with 2-nonynal afforded only poor yields. The reason for this low yield could be due to the instability of this type of polyfunctionalized and conjugated system. As an alternative, we considered an aldol coupling reaction as the key step. Deprotonation of (*R*)-4-hydroxymethyl-2,2-dimethyl-1,3-dioxolane with sodium hydride in DMF followed by the addition of the quantitatively mesylated alcohol **2** [11] gave the ether **4** in an acceptable yield of 58% (Scheme 1). Alkyne **4** was subjected to Hg(OAc)_2_ and PPTS-catalyzed hydration to afford ketone **6** in 60% isolated yield. The second acetylenic aldehyde moiety **11** was prepared by the alkylation of the propargyl alcohol **8 [12]** in the presence of *n*-BuLi and HMPA providing **9** in 82% yield which was then oxidized with Dess–Martin periodinane in CH_2_Cl_2_ [12]. The quantitively obtained aldehyde **11** was used without purification. Aldol condensation between the ketone **6** and the aldehyde **11** using *n*-BuLi in the presence of diisopropylamide [13] followed by overnight acid-promoted dehydration gave directly the deprotected ynenone **13** in 47% overall yield. This limited yield of **13** is no doubt due to the sensitivity of the starting aldehyde **11**.

Knowing that natural products **1a** and **1b** were unstable, it was expected that the synthetic intermediates would be as well. It was thus anticipated that the next steps involving selective reduction of the alkyne and the conjugated ketone together with the glycerol deprotection would generate yield issues. Actually, with the highly unsaturated system, the final hydrogenation turned out to be trickier than expected. Unsurprisingly then, classic conditions of partial alkyne hydrogenation with Lindlar catalyst [12,14] led to the complete reduction and giving the simple vinyl ketone. To overcome this problem, various solvents, reaction temperatures, and poisoning reagents were evaluated to limit reduction degree. The reaction at 0 °C in apolar solvent made it possible to limit over-reduction. The heptane/AcOEt 8:2 mixture of solvent was a good compromise to limit the solvent polarity while keeping a good solubility. It should also be noted that the starting alkyne does not react when using pyridine instead of quinoline. These developed conditions made it possible to increase the yield of the product from **15** to 87%. Without further purification, diene ketone **15** was then subjected to reduction by sodium borohydride to provide the epimeric mixture **1a + 1b** in 11% overall yield from **15**. Complete consumption of the starting material was observed by ^1^H NMR monitoring of both the alkyne hydrogenation and ketone reduction reaction mixtures. As the final product was sensitive to silica gel, it was purified by HPLC on a reversed-phase column with nevertheless substantial loss and consequent low yield.

Repeating the same reaction sequence starting from compounds **3** and **8** led to the analogous set of intermediates (**3**→**5**→**7**→**14**→**16**→**17**) and to the regioisomer **17** in comparable yields.

^1^H and ^13^C NMR spectra of the synthetic epimers **1a** + **1b** (m = 5, n = 7) corresponding to the proposed structure I were in accordance with those of the natural products named pericharaxins A (**1a**) and B (**1b**). The epimeric **17** (proposed structure II) showed clear signal differences with the natural epimers **1a** + **1b**, particularly with regard to the carbon chemical shifts (Table 1). The comparison of the spectra (Figure S29) made visible the chemical shift differences of C-3, C-4, and C-11 and thus allowed the complete attribution.

On the other hand, the ^1^H NMR spectra did not allow us to distinguish the presence of two epimers of the synthetic epimers **1a** and **1b**. To further identify the configuration of the natural product, the synthetic mixture **1a** + **1b** was subjected to chromatography on a chiral column to provide pure epimers **1a** and **1b**. Disappointingly, comparison of the NMR spectra of each pure compound did not show any obvious differences. This observation prompted us to run the natural product through the same chiral HPLC column used above for separation of the synthetic mixture. To our surprise, it was also found to be, like the synthetic product, epimeric (see SI, Figure S11). A very careful detailed analysis of the ^13^C NMR spectra nevertheless allowed identification a small difference of 0.07 ppm in the *δ*_C_ of the C-2’carbons of the epimers **1a** and **1b** (Figure S30).

**Figure 2:**
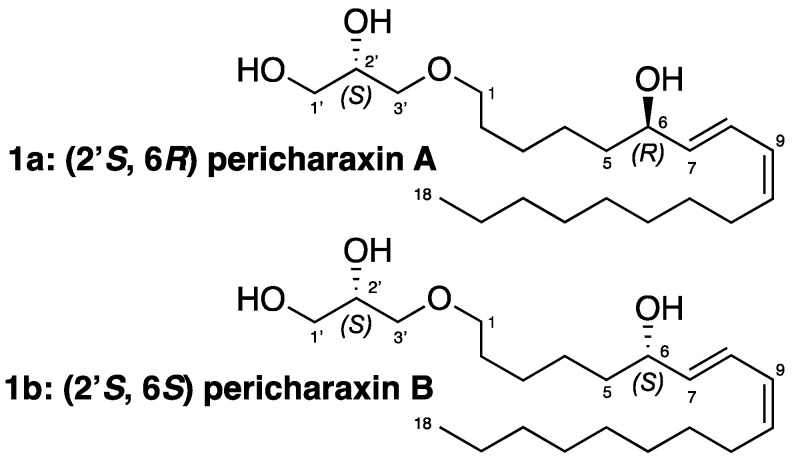
Absolute configuration of the two synthetic diastereoisomers (**1a**) and (**1b**) assigned by Mosher’s method.

The configuration at the C-2’ center of **1a** and **1b** being known from the starting material used for their synthesis (Scheme 1), the configuration at the C-6 chiral center was attributed through simple application of the Mosher method for one of the two separated isomers [15,16]. Thus, pure separated sample of epimer **1b** was treated with *R*(−)- and *S*(+)-methoxy-(trifluoromethyl)phenylacetyl chloride (MTPA-Cl) [15] to give the triesters **18** and **19** (Figure S2). The Mosher esters **18** and **19** were analyzed using 1D and 2D NMR spectroscopy [15]. The difference in the chemical shift (*δ^SR^*) was calculated for each of the analogous pairs of protons for both of (*S*)- and (*R*)-MTPA esters (Table S1, page S4) ((*R*)-MTPA ester: H2-5 *δ*_H_ 3.74; (*S*)-MTPA ester: H2-5 *δ*_H_ 5 3.71 Δ*δ* − 0.04). The positive values were assigned to the left side of the model (R1) and the negative values were placed on the right side (R2). By applying the Cahn–Ingold–Prelog system, the groups’ priorities were assigned as 1 (OH), 2 (C-7), 3 (C-5), and 4 (H), and the absolute configuration at C-6 was determined as (*S*) (Figure 2). The method was confirmed by the reassignment of the absolute configuration of C-2′ as (*S*). Therefore, the absolute configuration of **1b** is (2’S, 6S; ([α]^20^_D_ of −0.8 (c 0.90, CH_2_Cl_2_). Consequently, the configuration of **1a** (2’*S*, 6*R*; ([α]^20^_D_ of + 5.4 (c 0.95, CH_2_Cl_2_).

Compounds **1a**, **1b**, **2-16**, and **17**(**a** + **b**) were evaluated on a screening platform for endochondral differentiation activity using human type X collagen transcription activity in ATDC5 cells. Taking advantage of ATDC5 capacity to reproduce endochondral ossification and utilizing a promoter reporter assay for Col X, we developed a rapid in vitro screening platform to screen pro-endochondral differentiation small molecules including sponges’ extracted molecules and the newly synthesized compounds. There are spatial and temporal correlations between synthesis of type X collagen and occurrence of endochondral ossification. The expression of type X collagen is confined within hypertrophic chondrocytes and precedes the embark of endochondral bone formation [17]. Type X collagen facilitates endochondral ossification by regulating matrix mineralization and compartmentalizing matrix components.

We screened compounds **1a**, **1b**, **2**-**14**, and **17**(**a** + **b**) for endochondral differentiation capacity using the human ColX-promoter luciferase reporter (hColX- luc) transduced into the mouse chondrogenic line, ATDC5. The most active compounds **4** and **6** are able to stimulate ColX transcription, while compounds **1a**, **1b,** and **13** have the property to inhibit ColX transcription (see SI, Figure S31). The stimulation of ColX transcription means stimulation of endochondral ossification, involved in bone repair; reduction and inhibition of ColX transcription can be of interest to stop the final steps of endochondral ossification during chondrogenic differentiation of mesenchymal stem cells [18].

The above compounds were also assessed for their effects on the human cancer cell line HCT116 (colorectal cancer). The observed viability (>90%) showed that they have no cytotoxicity.

Glycerol ethers with various functionalities and substituents, originating from marine sponges and their associated algae such us *Spirastrella abata* [19,20] *Scopalina hapalia* [21] or of *Petrosia* sp. [22], *Niphates digitalis* [10], and *Petrosia* genus sponge, have previously been described [23]. Structurally similar to our pericharaxins A (**1a**) and B (**1b**), epimeric allylic alcohols of a monoalkeneglycerol were isolated from the associated Sponge-Alga *Haliclona cymaeformis/Ceratodictyon spongiosum* [6]. The latter compounds possess a 2(*S*) glycerol connected to a 16-carbon chain whereas our compound possesses an 18-carbo chain with an additional double bond as a part of a conjugated diene. In addition, the positioning of the system composed of the allylic alcohol function and the double bond is shifted by one carbon in the case of our pericharaxins A (**1a**) and B (**1b**). An interesting question concerns the biosynthetic origin of this epimeric compounds in sponges. Lipid peroxidation can be spontaneous or even non-enzymatic in the biological systems [24] giving rise to natural artifacts. The mechanism of this peroxidation requires a structure with a polyunsaturated system having at least one 1,4-cis-pentadiene unit. Peroxidation is then made possible by the malonic system allowing the formation of a bis-allylic radical which reacts with oxygen to give a peroxide. The bi-allylic radical can also rearrange to form another conjugated diene radical which in turn reacts with oxygen to give a monohydroxylated conjugated diene as proposed for leucettamol B [25]. In our case, this mechanism can be ruled out for two reasons: (1) no 1,4-pentadiene derivative of pericharaxins A (**1a**) and B (**1b**) were co-isolated or identified by LCMS, (2) two examples of epimers of the same type of allylic alcohol but containing only one double bond have been reported from sponges [6]. Such an allylic alcohol can only be formed enzymatically. Consequently, the mechanism of the formation of the epimers of pericharaxins and ceratodictyols, both glycerol ether derivatives, remains unexplained.

In mammalian cells, the biosynthesis of 1-*O*-alkylglycerol-based ether lipids is known and begins with the acylation of dihydroxyacetone phosphate (DHAP) with a long chain acyl-CoA ester [26]. The ether bond of glycerol ether lipids is formed by replacement of the O-acyl group by the O-alkyl group of a fatty alcohol. Interestingly, their occurrence in ocean waters and sediments has also been reported [27]. The existence of 1-*O*-monoalkyl glycerol ether lipids in sponge-associated algae suggests that labeling experiments may provide new insights into biosynthetic aspects and consequently their possible ecological role. Regarding their structural roles, some ether lipids are thought to function as antioxidants while other studies have suggested that they are involved in cell differentiation and signaling pathways and adipogenesis [7,8].

## 3. Materials and Methods

### 3.1. General Experimental Procedures

Optical rotations were measured using an Anton Paar MCP-300 polarimeter (Anton Paar France, Les Ulis, France) and are reported as follows: ${\left[\alpha \right]}_{D}^{T}$ °C (*c* = g/100 mL, solvent). Infrared data were obtained with a PerkinElmer Spectrum 65 spectrophotometer using an attenuated total reflectance (ATR) device (υ in cm^−1^). Nuclear magnetic resonance (^1^H and ^13^C NMR) spectra were recorded with a Bruker AVANCE 300 or a Bruker AVANCE 500 spectrometer. Proton NMR spectra are referenced from the residual proton in the NMR solvent (CDCl_3_: *δ*_H_ 7.26). Data are reported as follows: chemical shift (multiplicity (s = singlet, d = doublet, t = triplet, q = quartet, m = multiplet), coupling constant(s) in Hertz, integration). Carbon-13 NMR spectra are referenced from the carbon resonances of the solvent (CDCl_3_: *δ*_C_ 77.16). High resolution mass spectra (HRMS) were recorded on a Waters LCT Micromass spectrometer in positive electrospray ionization—time of flight (ESI-TOF) mode.

### 3.2. General Synthetic Experimental Procedures

All reactions were performed under inert atmosphere (argon), unless noted otherwise. Reactions conducted at elevated temperature were heated by immersion into a pre-heated oil bath. Medium-pressure liquid chromatography (MPLC) was performed on a Puriflash apparatus (Interchim, Montluçon, France) using the Interchim silica gel cartridge PF-50SIHP (Interchim, Montluçon, France). Analytical thin layer chromatography (TLC) was performed using aluminum plates pre-coated with 0.25 mm 230–400 mesh silica gel impregnated with a fluorescent indicator (254 nm). TLC plates were visualized by exposure to short wave ultraviolet light (254 nm) and irreversibly stained by treatment with a solution of sulfuric vanillin followed by heating with a heat gun. Organic solutions were concentrated at 29–35 °C on rotary evaporators capable of achieving a minimum pressure of ~10 mbar. Reported yields are unoptimized. All reagents were of commercial grade and were used without further purification unless otherwise stated. Commercially available anhydrous solvents were used for reactions conducted under inert atmosphere with the following exceptions: dichloromethane, tetrahydrofuran, and dimethylformamide were purified on a Pure Solv MD5 solvent purification system. All solvents and chemicals were purchased from Sigma-Aldrich or Fluorochem.

### 3.3. Animal Material

The sponge was collected by hand using SCUBA in Wallis and Futuna, during the sampling cruise Wallis 2018 aboard the R/V Alis [5], in the Wallis Island lagoon (13°16.177’ S, 176°08.120’ W) at a depth of 15 m, during July 2018. The collected sponges were immediately frozen after collection. A voucher sample has been deposited at the Queensland Museum (Brisbane, Australia) under the access number G339027 and was identified by Dr. Merrick Ekins as *Pericharax heteroraphis* Poléjaeff 1883. The sponge was deep frozen on board until work up. It was then ground, freeze-dried, and extracted.

### 3.4. Extraction and Isolation

The freeze-dried sponge sample of *Pericharax heteroraphis* (93 g) was extracted three times by ASE at 40 °C under pressure (100 bar) with a mixture of CH_2_Cl_2_/MeOH 1:1. The extracts were combined and dried under reduced pressure to afford a brown residue (6 g) and 5.5 g of this residue was partitioned between *n*-BuOH and H_2_O. The butanolic extract (1.4 g) was fractionated by reversed-phase C18 flash chromatography, eluting successively with solvents (H_2_O; H_2_O/CH_3_CN gradient; CH_3_CN; CH_2_Cl_2_; THF) to afford fractions F1 to F3.

Fraction F2 (H_2_O/CH_3_CN gradient) was purified by preparative reversed-phase HPLC (column: Waters Sunfire C18, 19 mm × 150 mm, 5 μm, H_2_O + 0.1% formic acid/CH_3_CN + 0.1% formic acid) to obtain 3.2 mg of the glycerol ether **1**.

*Pericharaxin* (1a + 1b)**:** 3.2 mg; UV (MeOH) λ_max_ (log ε) 229 (0.38); ^1^H NMR (500 MHz, CDCl_3_) *δ* ppm 6.49 (1H, dd, *J* = 15.2, 11.2 Hz, H-8), 5.98 (1H, t, *J* = 10.9 Hz, H-9), 5.67 (1H, dd, *J* = 15.2, 6.7 Hz, H-7), 5.44 (1H, dt, *J* = 10.9, 7.6 Hz, H-10), 4.17 (1H, dt, *J* = 6.7, 6.7 Hz, H-6), 3.87 (1H, dddd, *J* = 5.1, 3.8, 3.5, 3.4 Hz, H-2’), 3.72 (1H, dd, *J* = 11.2, 3.5 Hz, H-3’a), 3.65 (1H, dd, *J* = 11.2, 5.1 Hz, H-3’b), 3.54 (1H, dd, *J* = 9.6, 3.8 Hz, H-1’a), 3.52 (1H, dd, *J* = 9.6, 3.4 Hz, H-1’b), 3.47 (2H, dt, *J* = 6.6, 4.5 Hz, H-1), 2.18 (2H, tt, *J* = 7.6, 6.9 Hz, H-11), 1.57 (2H, dt, *J* = 13.5, 6.6 Hz, H-2), 1.53 (2H, dt, *J* = 12.9, 5.1 Hz, H-5), 1.39 (2H, t, *J* = 6.9 Hz), H-12), 1.30 (14H, 1.27–1.34, H-3, H-4 and H-13 to 17), 0.89 (3H, t, *J* = 6.6 Hz, H-18); ^13^C NMR (500 MHz, CDCl_3_) *δ* ppm 136.0 (C-7), 133.4 (C-10), 127.9 (C-9), 125.9 (C-8), 73.1 (C-6), 72.7 (C-1’), 71.9 (C-1), 70.6 (C-2’), 64.4 (C-3’), 37.4 (C-5), 31.9 (C-16), 29.7 (C-13), 29.6 (C-14), 29.4 (C-2), 29.3 (C-12), 27.8 (C-11), 29.1 (C-15), 27.8 (C-11), 26.1 (C-4), 25.3 (C-3), 22.7 (C-17), 14.2 (C-18); HRESIMS *m*/*z* 379.2799 [M + Na]^+^ (calcd for C_21_H_40_O_4_Na 379.2824), 339.2858 [M + H − H_2_O]^+^, 247.2405 [M + H − C_3_H_8_O_3_]^+^.

### 3.5. Synthesis of Pericharaxin (**1**)

*(R)-4-((Hept-6-yn-1-yloxy)methyl)-2,2-dimethyl-1,3-dioxolane* (**4**): Prepared following the literature procedure described in the literature [9]: (*R*)-4-hydroxymethyl-2,2-dimethyl-1,3-dioxolane (4.3 g, 32.5 mmol) was dissolved in dimethylformamide (100 mL) at 0 °C under an inert atmosphere. Sodium hydride (60% wt, 1.56 g, 35 mmol) was added in several portions. The reaction mixture was allowed to stir 40 min at this temperature. 6-Heptyn-1-ol 2 was mesylated as described in the literature [11] and the resulting mesylate (6.8 g, 35.7 mmol) in dimethylformamide (20 mL) was added. The reaction mixture was allowed to stir overnight at room temperature. The reaction was quenched by the addition of a saturated aqueous solution of ammonium chloride and extracted 3 times with diethyl ether. The combined organic layers were dried with MgSO_4_, filtered, and concentrated under reduced pressure. The crude product was purified by flash chromatography (elution by gradient heptane/EtOAc 100:0 to 60:40), to give **4** as a light-yellow oil (4.7 g, 58%). [α]_D_^20^ −13.4 (*c* 1.75, CH_2_Cl_2_); IR (neat) υ_max_ 3286, 2986, 2936, 2863, 2118, 1456 cm^−1^; ^1^H NMR (500 MHz, CDCl_3_) *δ* ppm 4.23 (quint, *J* = 6.1 Hz, 1H), 4.02 (dd, *J* = 8.2, 6.6 Hz, 1H), 3.70 (dd, *J* = 8.2, 6.6 Hz, 1H), 3.38–3.51 (m, 4H), 2.03 (dt, *J* = 2.5, 6.8 Hz, 2H), 2.16 (td, *J* = 7.0, 2.6 Hz, 2H), 1.91 (t, *J* = 2.6 Hz, 1H), 1.48–1.60 (m, 4H), 1.41–1.46 (m, 2H), 1.39 (s, 3H), 1.33 (s, 3H); ^13^C NMR (500 MHz, CDCl_3_) *δ* ppm 109.4, 84.4, 74.8, 71.9, 71.6, 68.3, 67.0, 29.1, 28.3, 26.8, 25.5, 25.3, 18.4; HRESIMS *m*/*z* 227.1647 [M + H]^+^ (calcd for C_12_H_23_O_3_ 227.1647).

*®-4-((Hex-5-yn-1-yloxy)methyl)-2,2-dimethyl-1,3-dioxolane* (**5**): Prepared following the literature procedure described in the literature [9]: (*R*)-4-hydroxymethyl-2,2-dimethyl-1,3-dioxolane (1.7 g, 12.9 mmol) was dissolved in dimethylformamide (75 mL) at 0 °C under inert atmosphere. Sodium hydride (60% wt, 620 mg, 15.5 mmol) was added in several portions. The reaction mixture was allowed to stir 40 min at this temperature. 6-Hexyn-1-ol 2 was mesylated as described in the literature [11] and the resulting mesylate (2.5 g, 14.2 mmol) in dimethylformamide (20 mL) was added. Reaction mixture was allowed to stir overnight at room temperature. The reaction was quenched by the addition of a saturated aqueous solution of ammonium chloride and extracted 3 times with diethyl ether. The combined organic layers were dried with MgSO_4_, filtered, and concentrated under reduced pressure. The crude product was purified by flash chromatography (elution by gradient heptane/EtOAc 100:0 to 60:40), to give 5 as a clear oil (1.62 g, 54%). ^1^H NMR (500 MHz, CDCl_3_) *δ* ppm 4.24 (quint, *J* = 6.1 Hz, 1H), 4.04 (dd, *J* = 8.2, 6.5 Hz, 1H), 3.72 (dd, *J* = 8.3, 6.5 Hz, 1H), 3.39–3.54 (m, 4H), 2.03 (dt, *J* = 2.5, 6.8 Hz, 2H), 1.93 (t, *J* = 2.5 Hz, 1H), 1.69 (2t, *J* = 6.6, 6.3 Hz,2H), 1.60 (t, *J* = 6.8 Hz, 1H), 1.57 (td, *J* = 2.5, 7.3 Hz, 2H), 1.41 (s, 3H), 1.35 (s, 3H).

*(R)-7-((2,2-Dimethyl-1,3-dioxolan-4-yl)methoxy)heptan-2-one* (**6**): To a stirred solution of (*R*)-4-((hept-6-yn-1-yloxy)methyl)-2,2-dimethyl-1,3-dioxolane (**4**) (4.7 g, 20.8 mmol) in tetrahydrofurane (100 mL, pH 7 buffer 9:1) at 0 °C were added pyridinium *p*-toluenesulfonate (8.7 g, 35 mmol) and mercuric acetate (734 mg, 2.3 mmol). The reaction mixture was warmed to 50 °C and stirred 5 h. The reaction was quenched by the addition of a saturated solution of sodium bicarbonate and extracted 3 times with ethyl acetate. The combined organic extracts were dried with MgSO_4_, filtered, and concentrated under reduced pressure. The crude product was purified by flash chromatography (elution by gradient heptane/EtOAc 100:0 to 50:50), to give **6** as a light-yellow oil (2.37 g, 60%). ${\left[\alpha \right]}_{20}^{D}$ −8.5 (*c* 3.37, CH_2_Cl_2_); IR (neat) υ_max_ 2987, 2935, 2864, 1714, 1455 cm^−1^; ^1^H NMR (500 MHz, CDCl_3_) *δ* ppm 4.21 (quint, *J* = 6.0 Hz, 1H), 4.01 (dd, *J* = 8.2, 6.6 Hz, 1H), 3.67 (dd, *J* = 8.2, 6.6 Hz, 1H), 3.35–3.48 (m, 4H), 2.39 (t, *J* = 7.4 Hz, 2H), 2.08 (s, 3H), 1.54 (m,2H), 1.37 (s, 3H), 1.32 (s, 3H); ^13^C NMR (500 MHz, CDCl_3_) *δ* ppm 208.9, 109.4, 74.8, 71.9, 71.5, 66.9, 43.6, 29.9, 29.4, 26.8, 25.7, 25.4, 23.6; HRESIMS *m*/*z* 245.1748 [M + H]^+^ (calcd for C_13_H_25_O_4_ 245.1753).

*(R)-6-((2,2-Dimethyl-1,3-dioxolan-4-yl)methoxy)hexan-2-one* (**7**): To a stirred solution of (*R*)-4-((hex-5-yn-1-yloxy)methyl)-2,2-dimethyl-1,3-dioxolane (**5**) (4.9 g, 23 mmol) in tetrahydrofurane (100 mL, pH 7 buffer 9:1) at 0 °C were added pyridinium *p*-toluenesulfonate (8.7 g, 35 mmol) and mercuric acetate (734 mg, 2.3 mmol). The reaction mixture was warmed to 50 °C and stirred 5 h. The reaction was quenched by the addition of a saturated solution of sodium bicarbonate, and extracted 3 times with ethyl acetate. The combined organic extracts were dried with MgSO_4_, filtered, and concentrated under reduced pressure. The crude product was purified by flash chromatography (elution by gradient heptane/EtOAc 100:0 to 50:50), to give **7** as a yellow oil (2.3 g, 43%). [α]_D_^20^ −17.9 (*c* 0.8, CH_2_Cl_2_); IR (neat) υ_max_ 2986, 2926, 28,577, 1714, 1456 cm^−1^; ^1^H NMR (500 MHz, CDCl_3_) *δ* ppm 4.25 (quint, *J* = 6.0 Hz, 1H), 4.04 (dd, *J* = 8.2, 6.6 Hz, 1H), 3.72 (dd, *J* = 8.2, 6.6 Hz, 1H), 3.38–3.54 (m, 4H), 2.45 (t, *J* = 7.0 Hz, 2H), 2.13 (s, 3H), 1.54–1.70 (m, 4H), 1.41 (s, 3H), 1.35 (s, 3H); ^13^C NMR (500 MHz, CDCl_3_) *δ* ppm 208.1, 108.9, 74.5, 71.6, 71.0, 66.5, 43.0, 29.5, 28.7, 26.5, 25.1, 20.2; HRESIMS *m*/*z* 231.1528 [M + H]^+^ (calcd for C_12_H_23_O_4_ 231.1596).

*Undec-2-yn-1-ol* (**9**): Prepared according to the literature from propargyl alcohol (5.5 g, 82%) [12].

*Dodec-2-yn-1-ol* (**10**): Prepared according to the literature from propargyl alcohol (4.1 g, 56%) [12].

*Undec-2-ynal* (**11**): To a cooled solution of undec-2-yn-1-ol (**9**) (4.9 g, 29.2 mmol) in dry dichloromethane (100 mL) was added Dess–Martin periodinane (11.8 g, 28 mmol) at 0 °C under inert atmosphere. The mixture was stirred for 30 min at the same temperature and filtered through a pad of celite using ethyl acetate as eluent. The filtered solution was concentrated and purified by flash chromatography (elution by a gradient heptane/EtOAc 100:0 to 75:25) to give **11** (4.9 g, quant.) as a light-yellow oil. IR (neat) υ_max_ 2925, 2855, 2200, 1685 cm^−1^; ^1^H NMR (500 MHz, CDCl_3_) *δ* ppm 9.17 (s, 1H), 2.40 (t, *J* = 7.0, Hz, 2H), 1.59 (tt, *J* = 7.2, 7.0 Hz, 2H), 1.40 (m, 2H), 1.28 (m, 8H), 0.88 (t, *J* = 6.6 Hz, 3H); ^13^C NMR (500 MHz, CDCl_3_) *δ* ppm 177.5, 99.7, 81.8, 31.9, 29.2, 29.1, 28.9, 27.7, 22.7, 19.2, 14.2; HRESIMS *m*/*z* 167.1427 [M + H]^+^ (calcd for C_11_H_19_O 167.1436).

*Dodec-2-ynal* (**12**): To a cooled solution of dodec-2-yn-1-ol (**10**) (3.5 g, 19 mmol) in dry dichloromethane (100 mL) was added Dess–Martin periodinane (7.7 g, 18 mmol) at 0 °C under an inert atmosphere. The mixture was stirred for 30 min at the same temperature and filtered through a pad of celite using ethyl acetate as eluent. The filtered solution was concentrated and purified by flash chromatography (elution by a gradient heptane/EtOAc 100:0 to 75:25) to give **12** (2.95 g, 86%) as a colorless oil. ^1^H NMR (500 MHz, CDCl_3_) *δ* ppm 9.18 (s, 1H), 2.41 (t, *J* = 7.2, Hz, 2H), 1.60 (tt, *J* = 7.3, 7.2 Hz, 2H), 1.41 (m, 2H), 1.28 (m, 12H), 0.88 (t, *J* = 6.6 Hz, 3H); ^13^C NMR (500 MHz, CDCl_3_) *δ* ppm 177.3, 99.5, 81.9, 32.0, 29.5, 29.4, 29.1, 28.9, 27.7, 22.7, 19.2, 14.2.

*(S,E)-1-(2,3-dihydroxypropoxy)octadec-7-en-9-yn-6-one* (**13**): LDA was prepared in situ: diisopropylamine (855 μL, 6.1 mmol) was dried on MgSO_4_ under an inert atmosphere and was dissolved in tetrahydrofuran (3 mL) at −78 °C. *n*-Butyllithium (5.1 mL, 6.4 mmol) was added turning the reaction mixture light yellow. Freshly prepared lithium diisopropylamide was dissolved in tetrahydrofuran (10 mL) at −78 °C and (*R*)-7-((2,2-dimethyl-1,3-dioxolan-4-yl)methoxy)heptan-2-one (**6**) (1.5 g, 6.1 mmol) was added dropwise. The reaction mixture was allowed to stir 5 min. Undec-2-ynal (**11**) (1.5 g, 9.2 mmol) was then added dropwise and the reaction mixture was allowed to warm to room temperature and to stir 25 min. The reaction was quenched by the addition of a saturated aqueous solution of ammonium chloride and extracted 3 times with diethyl ether. The combined organic layers were dried with MgSO_4_, filtered, and concentrated under reduced pressure to provide 3.8 g of crude product. The obtained acetonide was not purified and was used directly in the next step.

To the crude acetonide (2.5 g, 6.1 mmol) in THF/H_2_O 5:1 (60 mL) was added hydrochloric acid (2.9 mL, 30.5 mmol, 37% wt). The reaction mixture was warmed to 65 °C and allowed to stir for 12 h. The reaction was quenched by the addition of a saturated aqueous solution of sodium bicarbonate and extracted 3 times with dichloromethane. The combined organic extracts were dried with MgSO_4_, filtered, and concentrated under reduced pressure. The crude product was purified by flash chromatography (elution by gradient heptane/EtOAc 80:20 to 20:80), to give **13** as a white amorphous solid (1 g, 47% over two steps). ${\left[\alpha \right]}_{20}^{D}$ + 2.1 (*c* 0.75, CH_2_Cl_2_); IR (neat) υ_max_ 3353, 2925, 2855, 2211, 1685, 1460 cm^−1^; ^1^H NMR (500 MHz, CDCl_3_) *δ* ppm 6.65 (dt, *J* = 16.1, 2.3 Hz, 1H), 6.44 (d, *J* = 16.1 Hz, 1H), 3.86 (m, 1H), 3.72 (dd, *J* = 11.3, 3.9 Hz, 1H), 3.65 (dd, *J* = 11.1, 5.2 Hz, 1H), 3.46–3.56 (m, 4H), 2.54 (t, *J* = 7.2 Hz, 2H), 2.38 (td, *J* = 7.0, 2.2 Hz, 2H), 1.60 (m, 6H), 1.34–1.43 (m, 4H), 1.28 (s, 8H), 0.89 (t, *J* = 7.0 Hz, 3H); ^13^C NMR (500 MHz, CDCl_3_) *δ* ppm 199.5, 136.6, 124.3, 102.2, 78.6, 72.6, 71.5, 70.6, 64.3, 40.8, 31.9, 29.4, 29.3, 29.19, 29.0, 28.5, 25.9, 23.8, 22.8, 20.0, 14.2; HRESIMS *m*/*z* 353.2633 [M + H]^+^ (calcd for C_21_H_37_O_4_ 353.2692).

*1-(((R)-2,2-Dimethyl-1,3-dioxolan-4-yl)methoxy)-7-hydroxyoctadec-8-yn-5-one* (**14′**): LDA was prepared in situ: diisopropylamine (175 μL, 1.25 mmol) was dried on MgSO_4_ under an inert atmosphere and was dissolved in tetrahydrofurane (3 mL) at −78 °C. *n*-Butyllithium (1.2 mL, 1.31 mmol) was added turning the reaction mixture turned light yellow. Freshly prepared Lithium diisopropylamide was dissolved in tetrahydrofurane (5 mL) at −78 °C and (*R*)-6-((2,2-dimethyl-1,3-dioxolan-4-yl)methoxy)hexan-2-one (**7**) (230 mg, 1 mmol) was added dropwise. The reaction mixture was allowed to stir for 5 min. Dodec-2-ynal (**12**) (227 mg, 1.25 mmol) was then added dropwise and the reaction mixture was allowed to warm to room temperature and to stir for 25 min. The reaction was quenched by the addition of a saturated aqueous solution of ammonium chloride and extracted 3 times with diethyl ether. The combined organic layers were dried with MgSO_4_, filtered, and concentrated under reduced pressure. The crude product was purified by flash chromatography (elution by gradient heptane/EtOAc 100:0 to 50:50), to give **14**’ as a white amorphous solid (360 mg, 70%). ${\left[\alpha \right]}_{20}^{D}$ +1.2 (*c* 0.75, CH_2_Cl_2_); IR (neat) υ_max_ 3380, 2924, 2855, 2204, 1706, 1657, 1592, 1458 cm^−1^; ^1^H NMR (500 MHz, CDCl_3_) *δ* ppm 4.77 (m, 1H, H7), 4.25 (quint, *J* = 6.0 Hz, 1H, H2’), 4.04 (dd, *J* = 8.2, 6.5 Hz, 1H, H3’), 3.72 (dd, *J* = 8.2, 6.5 Hz, 1H, H3’), 3.39–3.52 (m, 4H, H1’ & H1), 2.85 (dd, *J* = 17.0, 7.9 Hz, 1H, H6), 2.75 (dd, *J* = 17.0, 3.7 Hz, 1H, H6), 2.45 (t, *J* = 7.0 Hz, 2H), 2.18 (m, 2H), 1.62–1.68 (m, 2H), 1.56–1.61 (m, 2H), 1.42 (s, 3H), 1.36 (s, 3H), 1.26 (s, 14H), 0.88 (t, *J* = 6.6 Hz, 3H); ^13^C NMR (500 MHz, CDCl_3_) *δ* ppm 210.1, 109.5, 86.0, 79.7, 74.9, 72.0, 71.4, 67.0, 58.8, 49.8, 43.3, 32.0, 29.6, 29.4, 29.2, 29.0, 28.9, 28.7, 26.9, 25.5, 22.8, 20.3, 18.8, 14.2; HRESIMS *m*/*z* 411.3133 [M + H]^+^ (calcd for C_24_H_42_O_5_ 411.3110).

*(S,E)-1-(2,3-Dihydroxypropoxy)11ctadic-6-en-8-yn-5-one* (**14**): To acetonide **14**’ (360 mg, 0.9 mmol) in THF/H_2_O 5:1 (15 mL) was added hydrochloric acid (430 μL, 4.4 mmol, 37% wt). The reaction mixture was heated to 65 °C and allowed to stir for 12 h. The reaction was quenched by the addition of a saturated aqueous solution of sodium bicarbonate and extracted 3 times with dichloromethane. The combined organic extracts were dried with MgSO_4_, filtered, and concentrated under reduced pressure. The crude product was purified by flash chromatography (elution by gradient heptane/EtOAc 80:20 to 0:100), to give **14** as a white amorphous solid (125 mg, 40%). ${\left[\alpha \right]}_{20}^{D}$ + 0.71 (*c* 0.70, CH_2_Cl_2_); IR (neat) υ_max_ 3348, 2922, 2849, 2235, 1740, 1642, 1598 cm^−1^; ^1^H NMR (500 MHz, CDCl_3_) *δ* ppm 6.65 (dt, *J* = 16.1, 2.3 Hz, 1H), 6.44 (d, *J* = 16.1 Hz, 1H), 3.86 (m, 1H), 3.72 (dd, *J* = 11.1, 3.9 Hz, 1H), 3.65 (dd, *J* = 11.1, 5.2 Hz, 1H), 3.49 (m, 4H), 2.56 (t, *J* = 7.0 Hz, 2H), 2.38 (td, *J* = 7.1, 2.3 Hz, 2H), 1.70 (quint, *J* = 7.0 Hz, 2H), 1.53–1.63 (m, 4H), 1.34–1.43 (m,2H), 1.26 (s, 10H), 0.88 (t, *J* = 7.0 Hz, 3H); ^13^C NMR (500 MHz, CDCl_3_) *δ* ppm 199.3, 136.5, 124.4, 102.2, 78.6, 72.6, 71.4, 70.6, 64.3, 40.5, 32.0, 29.6, 29.4, 29.2, 29.1, 29.0, 28.5, 22.8, 20.8, 20.0, 14.2; HRESIMS *m*/*z* 353.2661 [M + H]^+^ (calcd for C_21_H_37_O_4_ 353.2692).

*(2S)-3-(((6E,8Z)-5-Hydroxyoctadeca-6,8-dien-1-yl)oxy)propane-1,2-diol* (**17**): To (*S*,*E*)-1-(2,3-dihydroxypropoxy)octadec-6-en-8-yn-5-one (**14**) (70 mg, 0.2 mmol) dissolved in heptane/EtOAc 80:20 (5 mL) at 0 °C was added quinoline (129 mg, 1 mmol) and Lindlar catalyst (42 mg, 0.02 mmol, 5% wt). The reaction mixture was allowed to stir under an H_2_ atmosphere for 1.5 h. The reaction mixture was filtered on celite, eluted with ethyl acetate, and concentrated. The crude product was dissolved in methanol (5 mL) at 0 °C and sodium borohydride (11 mg, 0.3 mmol) was added. The reaction mixture was allowed to stir until complete consumption of the starting material (30 min). The reaction was quenched by the addition of water, the excess methanol was evaporated, and the aqueous layer was extracted 3 times with ethyl acetate. The combined organic extracts were dried with MgSO_4_, filtered, and concentrated under reduced pressure. The crude product was purified by HPLC (preparative C18 Sunfire column, elution by gradient H_2_O/CH_3_CN 80:20 to 0:100), to give **17** as a colorless oil (6 mg, 8% over two steps). [α]_D_^20^ + 4.0 (*c* 0.65, CH_2_Cl_2_); IR (neat) υ_max_ 3351, 2925, 2853, 1653, 1459 cm^−1^; ^1^H NMR (500 MHz, CDCl_3_) *δ* ppm 6.50 (dd, *J* = 15.2, 11.2 Hz, 1H), 5.97 (t, *J* = 10.9 Hz, 1H), 5.66 (dd, *J* = 15.2, 6.7 Hz, 1H), 5.46 (dt, *J* = 10.9, 7.6 Hz, 1H), 4.18 (dt, *J* = 6.7, 6.7 Hz, 1H), 3.87 (m, 1H), 3.72 (dd, *J* = 11.4, 3.9 Hz, 1H), 3.65 (dd, *J* = 11.4, 5.1 Hz, 1H), 3.45–3.56 (m, 4H), 2.18 (q, *J* = 7.2 Hz, 2H), 1.54–1.66 (m, 2H), 1.44–1.53 (m, 2H), 1.35–1.42 (m, 2H), 1.27 (s, 14H), 0.89 (t, *J* = 6.6 Hz, 3H); ^13^C NMR (500 MHz, CDCl_3_) *δ* ppm 135.6, 133.4, 127.7, 126.2, 72.8, 72.6, 71.6, 70.6, 64.3, 37.0, 32.0, 29.8, 29.7, 29.6, 29.5, 29.4, 29.4, 27.9, 22.8, 22.1, 14.2.

Synthetic epimers *Pericharaxins* **A** (**1a**) and **B** (**1b**): To (*S*,*E*)-1-(2,3-dihydroxypropoxy)octadec-7-en-9-yn-6-one (**13**) (300 mg, 0.85 mmol) dissolved in heptane/EtOAc 8:2 (15 mL) at 0 °C was added quinoline (504 μL, 4.3 mmol) and Lindlar catalyst (179 mg, 0.09 mmol, 5% wt). The reaction mixture was allowed to stir under an H_2_ atmosphere for 1.5 h. The reaction mixture was filtered on celite, eluted with ethyl acetate, and concentrated. The crude product was dissolved in methanol (15 mL) at 0 °C and sodium borohydride (48.5 mg, 1.28 mmol) was added. Reaction mixture was allowed to stir until complete consumption of the starting material (30 min). The reaction was quenched by the addition of water, the excess methanol was evaporated, and the aqueous layer was extracted 3 times with ethyl acetate. The combined organic extracts were dried with MgSO_4_, filtered, and concentrated under reduced pressure. The crude product was purified by HPLC (preparative C18 Sunfire column, elution by gradient H_2_O/CH_3_CN 80:20 to 0:100), to give **1** as a yellow oil (32 mg, 10% over two steps). ${\left[\alpha \right]}_{20}^{D}$ +1.8 (*c* 0.62, CH_2_Cl_2_); IR (neat) υ_max_ 3360, 2950, 2855, 1460 cm^−1^; ^1^H NMR (500 MHz, CDCl_3_) *δ* ppm 6.49 (dd, *J =* 15.4, 11.0 Hz, 1H, H-8), 5.97 (t, *J* = 11.0 Hz, 1H, H-9), 5.66 (dd, *J* = 15.4, 6.7 Hz, 1H, H-7), 5.46 (dt, *J* = 11.0, 7.6 Hz, 1H, H-10), 4.16 (dt, *J* = 6.7, 6.4 Hz, 1H, H-6), 3.86 (m, 1H, H-2’), 3.72 (dd, *J* = 11.3, 3.8 Hz, 1H, H-3’a), 3.64 (dd, *J* = 11.3, 5.1 Hz, 1H, H-3’b), 3.44–3.55 (m, 4H, H-1’ and H-1), 2.18 (q, *J* = 7.2 Hz, 2H, H-11), 1.60 (m, 2H, H-2), 1.48–1.57 (m, 2H, H-5), 1.38–1.47 (m, 2H, H-12), 1.34–1.41 (m, 4H, H-3 and H-4), 1.24–1.32 (m, 10H, H-13 to H-17), 0.89 (t, *J* = 7.0 Hz, 3H, H-18); ^13^C NMR (500 MHz, CDCl_3_) *δ* ppm 135.7 (C-7), 133.3 (C-10), 127.7 (C-9), 126.1 (C-8), 72.9 (C-6), 72.6 (C-1’), 71.8 (C-1), 70.6 (C-2’), 64.4 (C-3’), 37.3 (C-5), 32.0 (C-16), 29.8 (C-13), 29.6 (C-14), 29.5 (C-2), 29.4 (C-15, C-12), 27.9 (C-11), 26.2 (C-4), 25.3 (C-3), 22.8 (C-17), 14.2 (C-18); HRESIMS *m*/*z* 379.2827 [M + Na]^+^ (calcd for C_21_H_40_O_4_Na 379.2824).

The two diastereoisomers were separated by chiral chromatography on an amylose tris(3-chlorophenylcarbamate) immobilized on 5 μm silica column (analytical 4.6 × 250 mm, 1 mL/min and preparative 10 × 250 mm, 4.2 mL/min; isocratic heptane/isopropanol 90:10) to give Pricharaxin A (**1a**) (R_T_ 16.9 min) and B (**1b**) (R_T_ 19.4 min).

*2’(S)-6(R)-Pericharaxin A* (**1a**): ${\left[\alpha \right]}_{20}^{D}$ + 5.4 (*c* 0.95, CH_2_Cl_2_); ^1^H NMR (500 MHz, CDCl_3_) *δ* ppm 6.50 (dd, *J* = 15.1, 11.0 Hz, 1H, H-8), 5.97 (t, *J* = 10.9 Hz, 1H, H-9), 5.66 (dd, *J* = 15.2, 6.9 Hz, 1H, H-7), 5.46 (dt, *J* = 10.9, 7.6 Hz, 1H, H-10), 4.17 (dt, *J* = 6.6, 6.6 Hz, 1H, H-6), 3.86 (m, 1H, H-2’), 3.72 (dd, *J* = 11.4, 3.9 Hz, 1H, H-3’a), 3.65 (dd, *J* = 11.4, 5.1 Hz, 1H, H-3’b), 3.54 (dd, *J* = 9.5, 3.7 Hz, 1H, H-1’a), 3.50 (dd, *J* = 9.5, 6.1 Hz, 1H, H-1’b), 3.47 (t, *J* = 6.2 Hz, 2H, H-1), 2.18 (q, *J* = 7.2 Hz, 2H, H-11), 1.58–1.63 (m, 2H, H-2), 1.49–1.58 (m, 2H, H-5), 1.35–1.42 (m, 6H, H3, H-4 and H-12), 1.23–1.33 (m, 10H, H-13 to H-17), 0.89 (t, *J* = 7.0 Hz, 3H, H-18); ^13^C NMR (500 MHz, CDCl_3_) *δ* ppm 135.8 (C-7), 133.4 (C-10), 127.7 (C-9), 126.1 (C-8), 72.9 (C-6), 72.7 (C-1’), 71.8 (C-1), 70.6 (C-2’), 64.4 (C-3’), 37.3 (C-5), 32.0 (C-16), 29.8 (C-13), 29.6 (C-2, C-14), 29.4 (C-12, C-15), 27.9 (C-11), 26.2 (C-4), 25.3 (C-3), 22.8 (C-17), 14.2 (C-18).

*2’(S)-6(S)-Pericharaxin B* (**1b**): [α]_D_^20^ −0.8 (*c* 0.90, CH_2_Cl_2_); ^1^H NMR (500 MHz, CDCl_3_) *δ* ppm 6.50 (dd, *J* = 15.1, 11.0 Hz, 1H, H-8), 5.97 (t, *J* = 10.9 Hz, 1H, H-9), 5.66 (dd, *J* = 15.2, 6.9 Hz, 1H, H-7), 5.46 (dt, *J* = 10.9, 7.6 Hz, 1H, H-10), 4.17 (dt, *J* = 6.6, 6.6 Hz, 1H, H-6), 3.86 (m, 1H, H-2’), 3.72 (dd, *J* = 11.4, 3.9 Hz, 1H, H-3’a), 3.65 (dd, *J* = 11.4, 5.1 Hz, 1H, H-3’b), 3.54 (dd, *J* = 9.5, 3.7 Hz, 1H, H-1’a), 3.50 (dd, *J* = 9.5, 6.1 Hz, 1H, H-1’b), 3.47 (t, *J* = 6.2 Hz, 2H, H-1), 2.18 (q, *J* = 7.2 Hz, 2H, H-11), 1.58–1.63 (m, 2H, H-2), 1.49–1.58 (m, 2H, H-5), 1.35–1.42 (m, 6H, H3, H-4 and H-12), 1.23–1.33 (m, 10H, H-13 to H-17), 0.89 (t, *J* = 7.0 Hz, 3H, H-18); ^13^C NMR (500 MHz, CDCl_3_) *δ* ppm 135.8 (C-7), 133.4 (C-10), 127.7 (C-9), 126.1 (C-8), 72.9 (C-6), 72.7 (C-1’), 71.8 (C-1), 70.6 (C-2’), 64.4 (C-3’), 37.3 (C-5), 32.0 (C-16), 29.8 (C-13), 29.6 (C-2, C-14), 29.4 (C-12, C-15), 27.9 (C-11), 26.2 (C-4), 25.3 (C-3), 22.8 (C-17), 14.2 (C-18).

*Mosher determination of the C-6 configuration for epimer (1b)* [15,16]: To a stirred solution of compound **1b** (1 mg, 0.003 mmol) in anhydrous pyridine (80 μL) at room temperature, DMAP (1 crystal) was added under an inert atmosphere. After 5 min, *R*-(-)-MTPA-Cl (4.3 mg, 0.017 mmol, 6 equiv.) was added. The reaction progress was monitored by TLC on silica gel (100% EtOAc). After 12 h, Pericharaxin (**1b**) was totally consumed. The pyridine solvent was evaporated, the crude product was dissolved in CH_2_Cl_2_, washed with water, and concentrated, to give the ®-MTPA-triesterified product (**18**). ^1^H NMR (500 MHz, CDCl_3_): See Table S1.

To a stirred solution of Pericharaxin B (**1b**) (1 mg, 0.003 mmol) in anhydrous pyridine (80 μL) at room temperature, DMAP (1 crystal) was added under an inert atmosphere. After 5 min, *S*-(-)-MTPA-Cl (4.3 mg, 0.017 mmol, 6 equiv.) was added. The reaction progress was monitored by TLC on silica gel (100% EtOAc). After 12 h, compound **1b** was totally consumed. The pyridine solvent was evaporated, the crude product was dissolved in dichloromethane, washed with water, and concentrated, to give the (*S*)-MTPA-triesterified product **19**. ^1^H NMR (500 MHz, CDCl_3_):See Table S1.

## Figures and Tables

**Table 1:** ^13^C NMR (125 MHz, CDCl_3_, δ ppm) comparison of natural and synthetic samples of epimeric compounds **1** (**1a** + **1b**) and **17** (**17a** + **17b**); chemical shift differences of C-3, C-4m and C-11 are in bold.

Position	Natural Epimers 1a + 1b	Synthetic Epimers 1a + 1b	Synthetic Epimers 17a + 17b
1	71.9, CH_2_	71.8, CH_2_	71.6, CH_2_
2	29.4, CH_2_	29.5, CH_2_	29.8, CH_2_
3	**26.1, CH_2_**	**26.2, CH_2_**	**22.1, CH_2_**
4	**25.3, CH_2_**	**25.3, CH_2_**	**37.0, CH_2_**
5	37.4, CH_2_	37.3, CH_2_	72.8, CH
6	73.1, CH	72.9, CH	135.6, CH
7	136.0, CH	135.7, CH	126.2, CH
8	125.9, CH	126.1, CH	127.7, CH
9	127.9, CH	127.7, CH	133.4, CH
10	133.4, CH	133.3, CH	27.9, CH_2_
11	**27.8, CH_2_**	**27.9, CH_2_**	**29.7, CH_2_**
12	29.3, CH_2_	29.4, CH_2_	29.6, CH_2_
13	29.7, CH_2_	29.8, CH_2_	29.5, CH_2_
14	29.6, CH_2_	29.6, CH_2_	29.4, CH_2_
15	29.1, CH_2_	29.2, CH_2_	29.4, CH_2_
16	31.9, CH_2_	32.0, CH_2_	32.0, CH_2_
17	22.7, CH_2_	22.8, CH_2_	22.8, CH_2_
18	14.2, CH_3_	14.2, CH_3_	14.2, CH_3_
1’	72.7, CH_2_	72.6, CH_2_	72.6, CH_2_
2’	70.6, CH	70.6, CH	70.6, CH
3’	64.4, CH_2_	64.4, CH_2_	64.3, CH_2_

## Data Availability

The data presented in this study are available in the provided Supporting Information and upon request from the corresponding authors.

## Supplementary Materials

The following supporting information can be downloaded at: https://www.mdpi.com/article/10.3390/md20100635/s1, Spectroscopic data, copies of ^1^H, ^13^C, NMR data and spectra, HPLC chromatograms and biological screening profile.
