# Supplementary material for: Isolation, Synthesis and Absolute Configuration of the Pericharaxins A and B, Epimeric Hydroxy-Polyene Glycerol Ethers from the Calcarean Sponge Pericharax heteroraphis
Source: Mar Drugs. 2022 Oct 11;20(10):635. doi: 10.3390/md20100635 (PMC9605606; doi:10.3390/md20100635)

Supporting Information

Table of Contents

Figure S1: UV chromatogram of fraction F2 containing pericharaxins A and B (**1a** + **1b**)..................4

Table S1. ^{1}H NMR Data of (S)- and (R)-MTPA-triesters and shifts comparison (500 MHz, $CDCl_3$). 5

Figure S2: Representation of the conformation of each MTPA esters and the resulting Δ^{SR} values (in ppm) for each of the protons...5

Table S2. ^{13}C NMR Data in δ ppm of natural and synthetic compounds **1a** + **1b** and compound **17a** + **17b** (125 MHz, $CDCl_3$). 6

Figure S3: ^{1}H NMR spectrum of natural pericharaxins A + B (**1a** + **1b**) (500 MHz, $CDCl_3$)...............7

Figure S4: ^{13}C NMR spectrum of natural pericharaxin A + B (**1a** + **1b**) (125 MHz, $CDCl_3$)................7

Figure S5: ^{1}H NMR spectrum of synthetic pericharaxins A + B (**1a** + **1b**) (500 MHz, $CDCl_3$)............8

Figure S6: ^{13}C NMR spectrum of synthetic pericharaxins A + B (**1a** + **1b**) (125 MHz, $CDCl_3$). 8

Figure S7: ^{1}H NMR spectrum of synthetic pericharaxin (**1a**) (500 MHz, $CDCl_3$)................................9

Figure S8: ^{13}C NMR spectrum of synthetic pericharaxin (**1a**) (125 MHz, $CDCl_3$). 9

Figure S9: ^{1}H NMR spectrum of synthetic pericharaxin (**1b**) (500 MHz, $CDCl_3$). 10

Figure S10: ^{13}C NMR spectrum of synthetic pericharaxin (**1b**) (125 MHz, $CDCl_3$)...........................10

Figure S11: Chiral HPLC chromatograms of natural and synthetic compounds comparison.........11

Figure S12: ^{1}H NMR spectrum of compound **4** (500 MHz, $CDCl_3$)..12

Figure S13: ^{13}C NMR spectrum of compound **4** (125 MHz, $CDCl_3$). 12

Figure S14: ^{1}H NMR spectrum of compound **5** (500 MHz, $CDCl_3$)..13

Figure S15: ^{1}H NMR spectrum of compound **6** (500 MHz, $CDCl_3$)..14

Figure S16: ^{13}C NMR spectrum of compound **6** (125 MHz, $CDCl_3$). 14

Figure S17: ^{1}H NMR spectrum of compound **7** (500 MHz, CDCl3)..15

Figure S18: ^{13}C NMR spectrum of compound **7** (125 MHz, $CDCl_3$). 15

Figure S19: ^{1}H NMR spectrum of compound **11** (300 MHz, $CDCl_3$)..16

Figure S20: ^{13}C NMR spectrum of compound **11** (75 MHz, $CDCl_3$). 16

Figure S21: ^{1}H NMR spectrum of compound **12** (500 MHz, $CDCl_3$)..17

Figure S22: ^{13}C NMR spectrum of compound **12** (125 MHz, $CDCl_3$). 17

Figure S23: ^{1}H NMR spectrum of compound **13** (300 MHz, $CDCl_3$)..18

Figure S24: ^{13}C NMR spectrum of compound **13** (75 MHz, $CDCl_3$). 18

Figure S25: ^{1}H NMR spectrum of compound **14'** (500 MHz, $CDCl_3$)..19

Figure S26: ^{1}H NMR spectrum of compound **14'** (125 MHz, $CDCl_3$)..19

Figure S27: ^{1}H NMR spectrum of compound **14** (500 MHz, $CDCl_3$)..20

Figure S28: ^{13}C NMR spectrum of compound **14** (125 MHz, $CDCl_3$). 20

Figure S29: NMR comparisons of natural product with the two synthetic products 21

Figure S30: ^{13}C NMR comparison of epimers **1a** (*S*,*R*) and **1b** (*S*,*S*) 22

Figure S31: results of the assessment of an ATDC5-based rapid screening platform using the human Col X (hCol X) proximal promoter activity for selection of natural compounds able to stimulate endochondral differentiation. hColX-promoter activity in murine ATDC5 chondrocyte line cultured under endochondral differentiation conditions as assessed by measurement of luminometric luciferase signals. 22

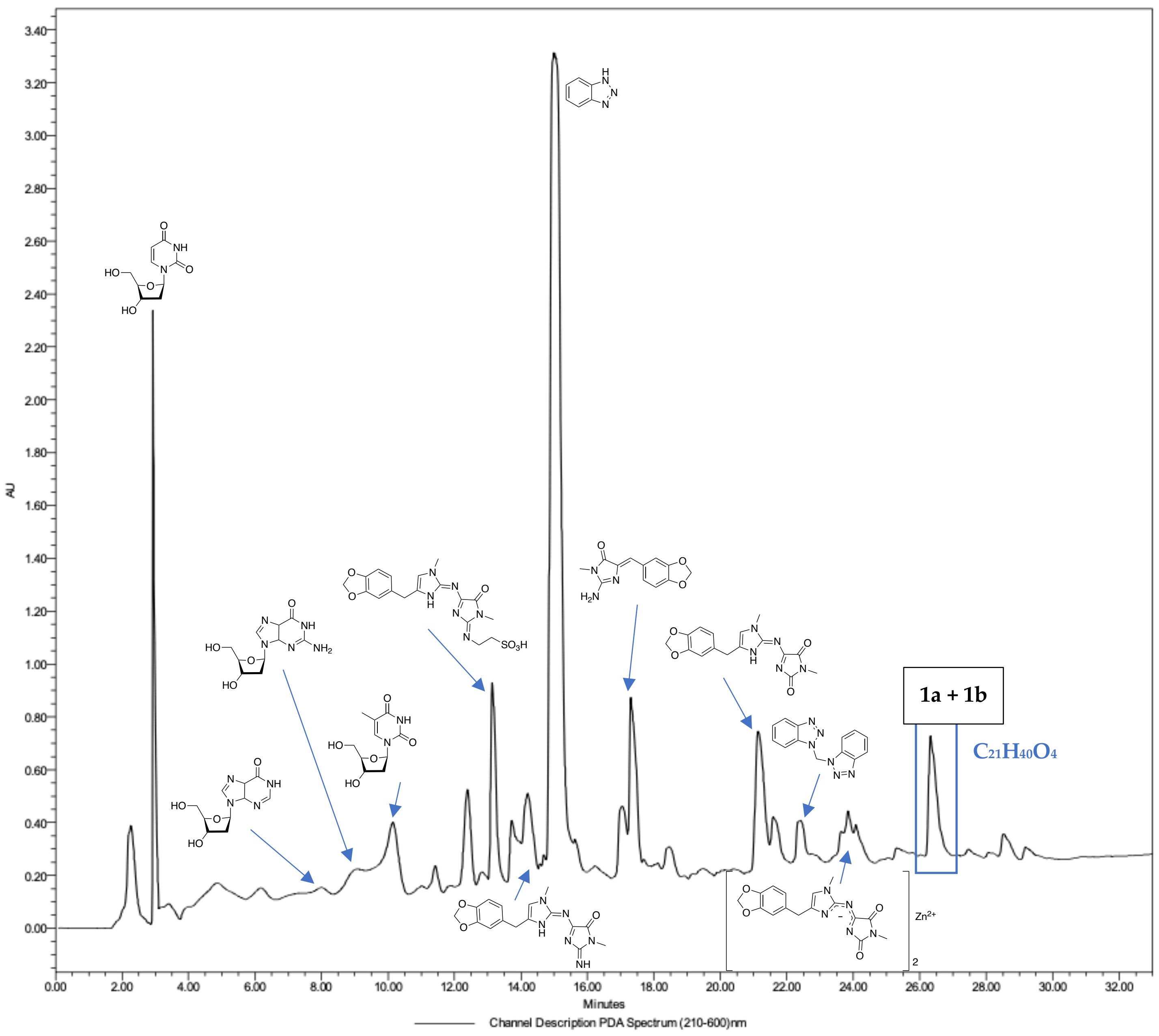

Figure S1: UV chromatogram of fraction F2 containing pericharaxins A and B (**1a** + **1b**)

Table S1. ^{1}H NMR Data of (*S*)- and (*R*)-MTPA-triesters and shifts comparison (500 MHz, $CDCl_3$).

N°	S-MTPA-triester (**18**) δ_H (ppm)	R-MTPA-triester (**19**) δ_H (ppm)	Δ^{SR}
1	3,33	3,27	0,06
2	1,45	1,44	0,01
3	1,26	1,32	-0,06
4	1,59	1,62	-0,03
5	1,68	1,72	-0,04
6	5,52	5,49	0,03
7	5,59	5,50	0,09
8	6,62	6,54	0,08
9	5,95	5,89	0,06
10	5,53	5,49	0,04
11	2,16	2,12	0,04
12	1,36	1,32	0,04
13-17	1,26	1,26	0
18	0,88	0,88	0
3′a	4,62	4,70	-0,08
3′b	4,35	4,41	-0,06
2′	5,46	5,42	0,04
1	3,57	3,46	0,11
OMe	3,54/3,59/3,43	3,52/3,46/3,38	

Figure S2: Representation of the conformation of each MTPA esters and the resulting Δ^{SR} values (in ppm) for each of the protons.

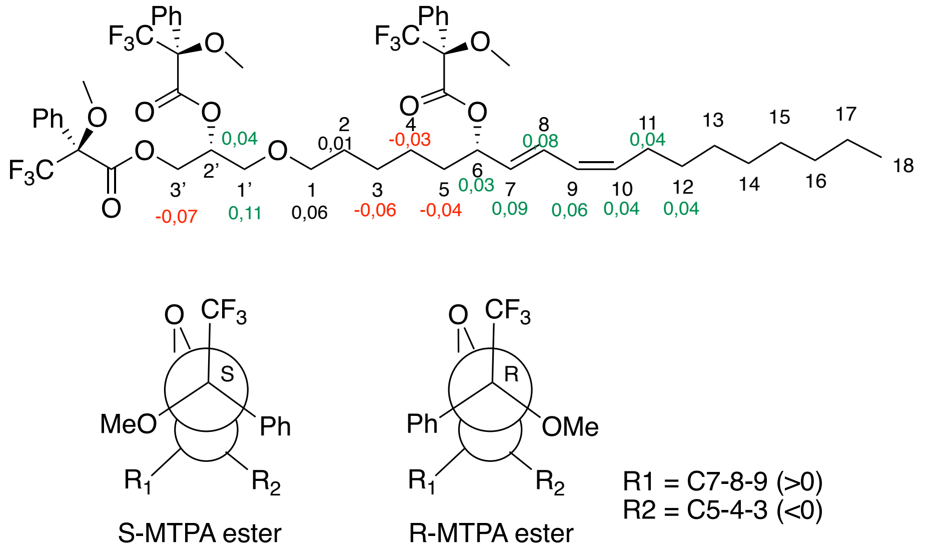

Table S2. ^{13}C NMR Data in δ ppm of natural and synthetic Compound **1a + 1b** and compound **17a + 17b** (125 MHz, $CDCl_3$).

N°	Natural compounds **1a + 1b**	Synthetic compounds **1a + 1b**	Synthetic compounds **17a + 17b**
1	71.9, CH_2	71.8, CH_2	71.6, CH_2
2	29.4, CH_2	29.5, CH_2	29.8, CH_2
3	**26.1, CH_2**	**26.2, CH_2**	**22.1, CH_2**
4	**25.3, CH_2**	**25.3, CH_2**	37.0, CH_2
5	37.4, CH_2	37.3, CH_2	72.8, CH
6	73.1, CH	72.9, CH	135.6, CH
7	136.0, CH	135.7, CH	126.2, CH
8	125.9, CH	126.1, CH	127.7, CH
9	127.9, CH	127.7, CH	133.4, CH
10	133.4, CH	133.3, CH	27.9, CH_2
11	27.8, CH_2	27.9, CH_2	29.7, CH_2
12	29.3, CH_2	29.4, CH_2	29.6, CH_2
13	29.7, CH_2	29.8, CH_2	29.5, CH_2
14	29.6, CH_2	29.6, CH_2	29.4, CH_2
15	29.1, CH_2	29.2, CH_2	29.4, CH_2
16	31.9, CH_2	32.0, CH_2	32.0, CH_2
17	22.7, CH_2	22.8, CH_2	22.8, CH_2
18	14.2, CH_3	14.2, CH_3	14.2, CH_3
1′	72.7, CH_2	72.6, CH_2	72.6, CH_2
2′	70.6, CH	70.6, CH	70.6, CH
3′	64.4, CH_2	64.4, CH_2	64.3, CH_2

Figure S3: ^{1}H NMR spectrum of natural pericharaxins A + B (**1a + 1b**) (500 MHz, $CDCl_3$).

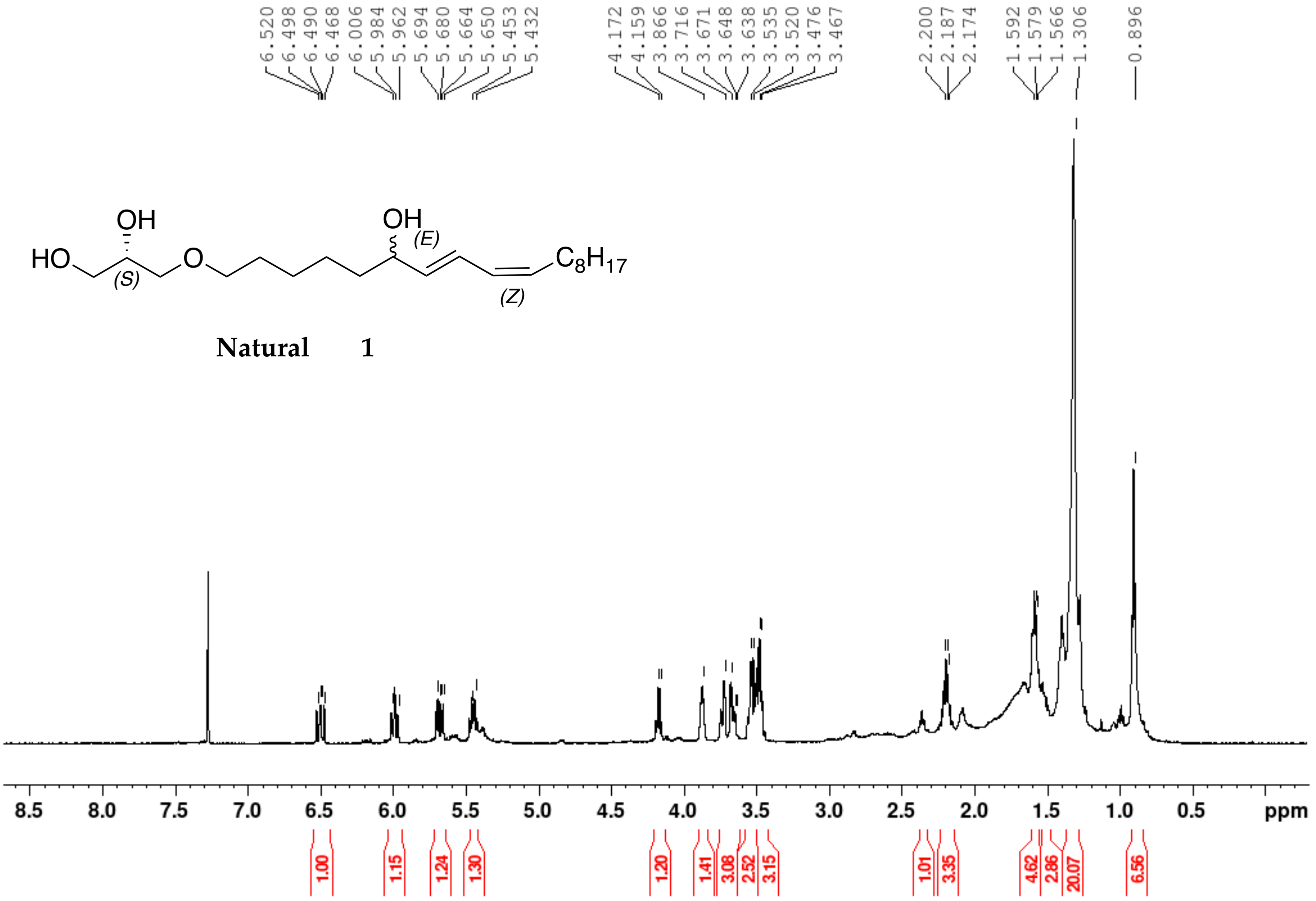

Figure S4: ^{13}C NMR spectrum of natural pericharaxin A + B (**1a + 1b**) (125 MHz, $CDCl_3$).

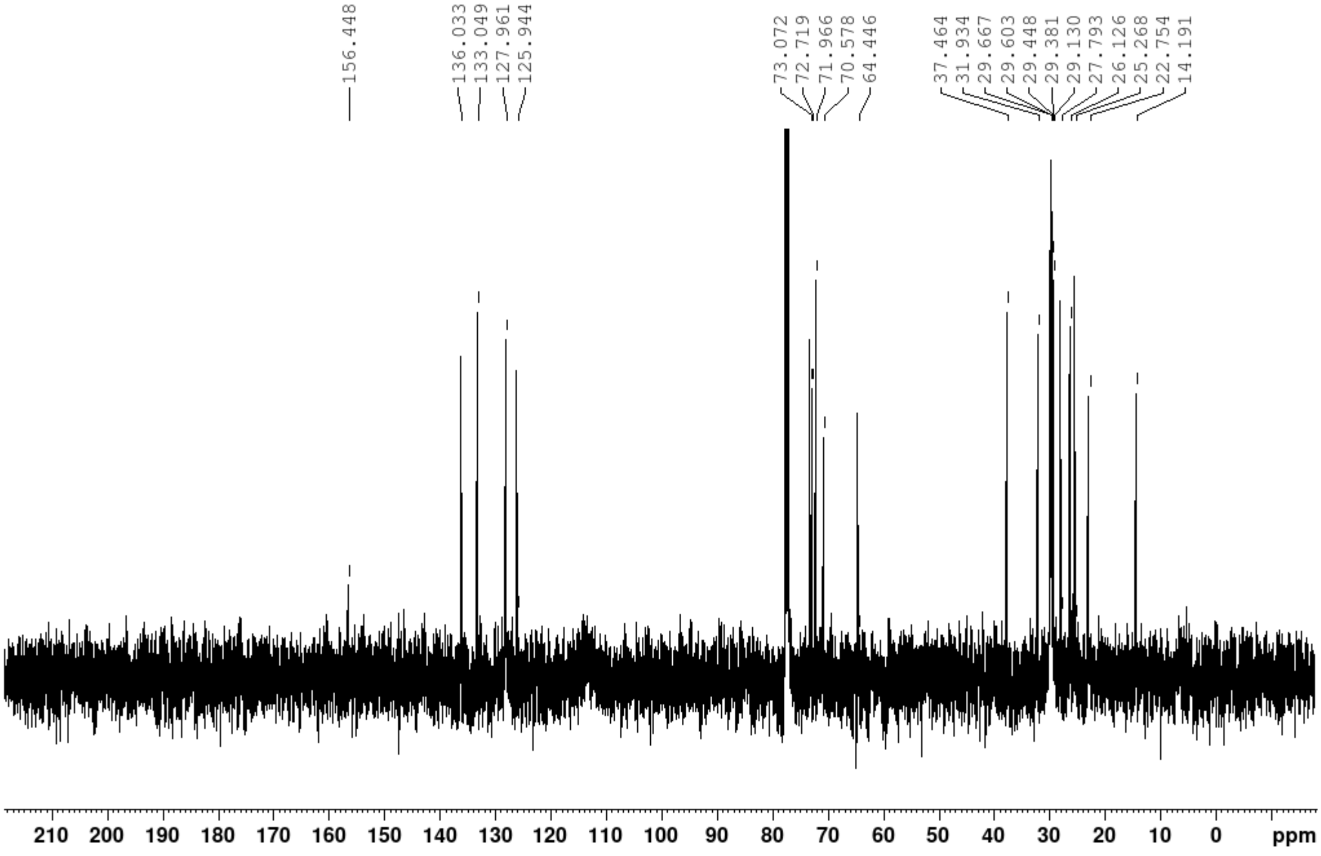

Figure S5: ^{1}H NMR spectrum of synthetic pericharaxins A + B (**1a + 1b**) (500 MHz, $CDCl_3$).

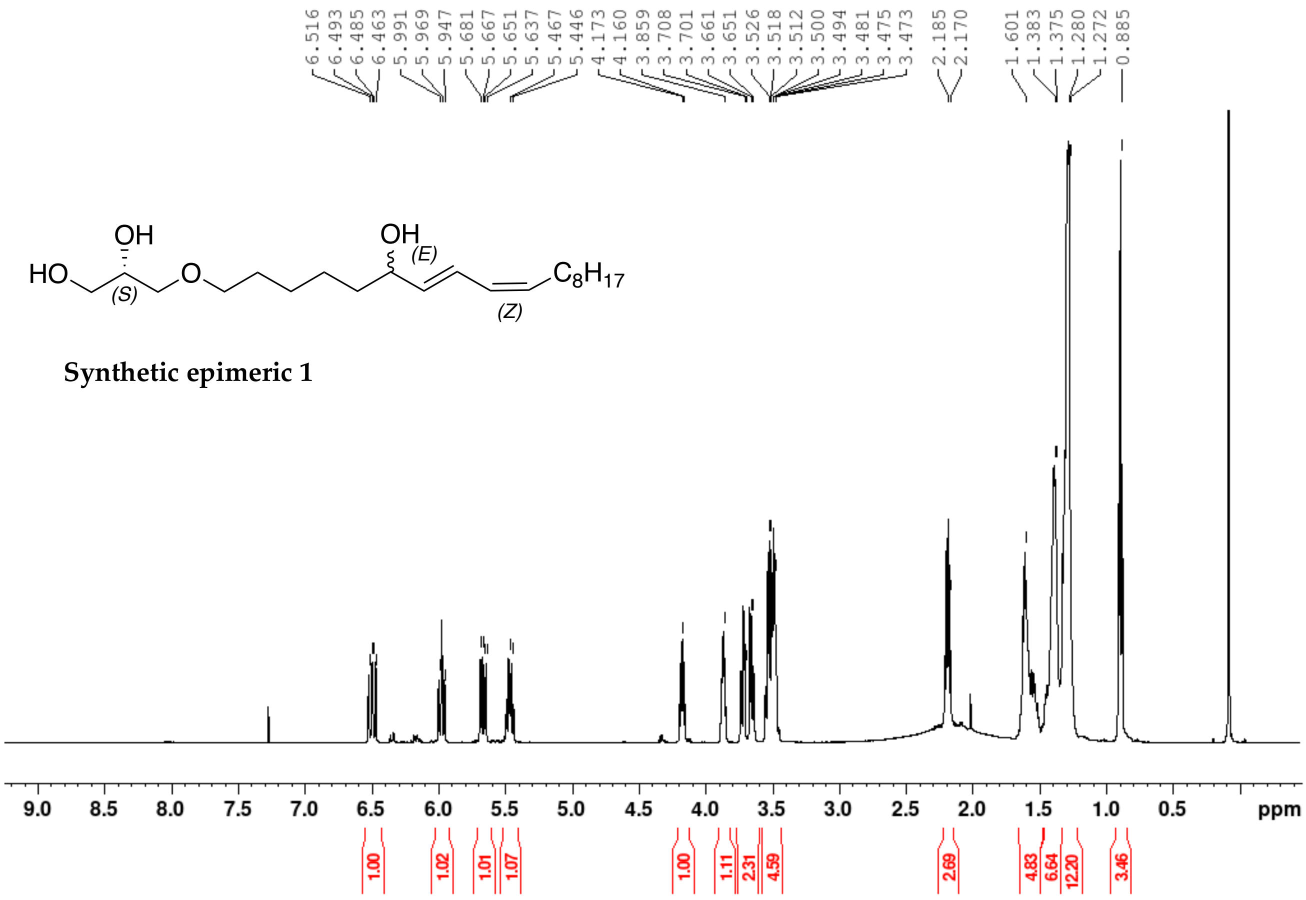

Figure S6: ^{13}C NMR spectrum of synthetic pericharaxins A + B (**1a + 1b**) (125 MHz, $CDCl_3$).

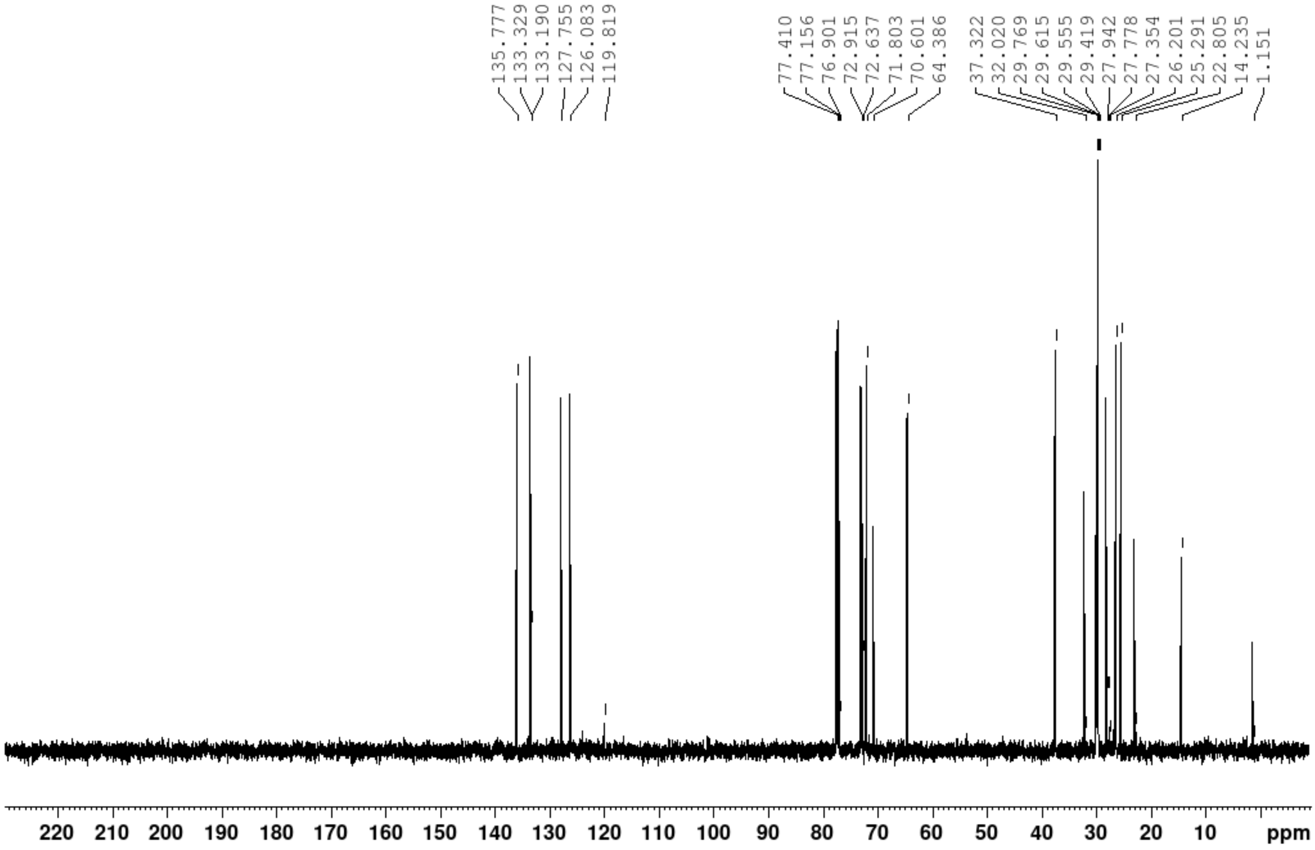

Figure S7: ^{1}H NMR spectrum of synthetic pericharaxin (**1a**) (500 MHz, $CDCl_3$).

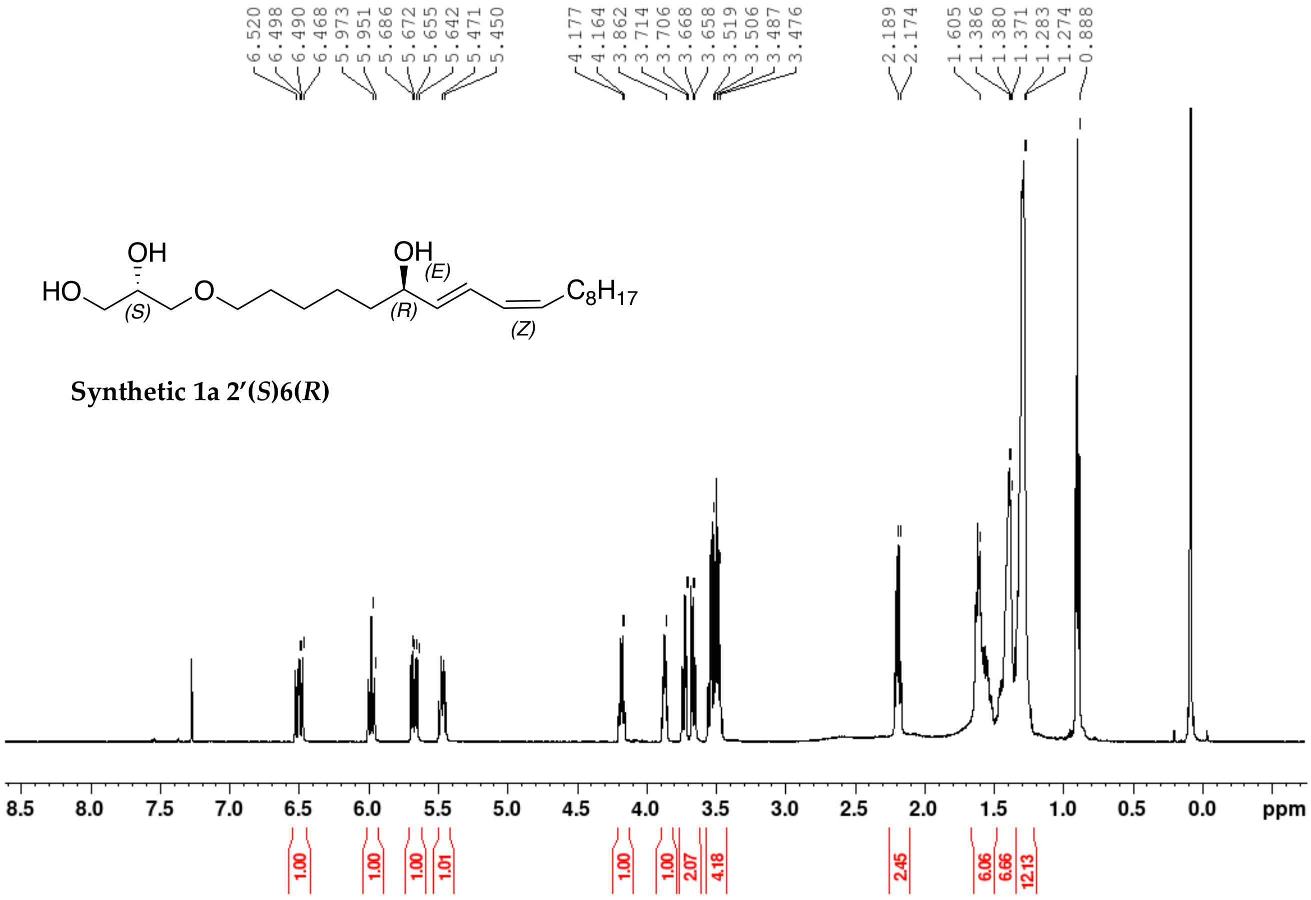

Figure S8: ^{13}C NMR spectrum of synthetic pericharaxin (**1a**) (125 MHz, $CDCl_3$).

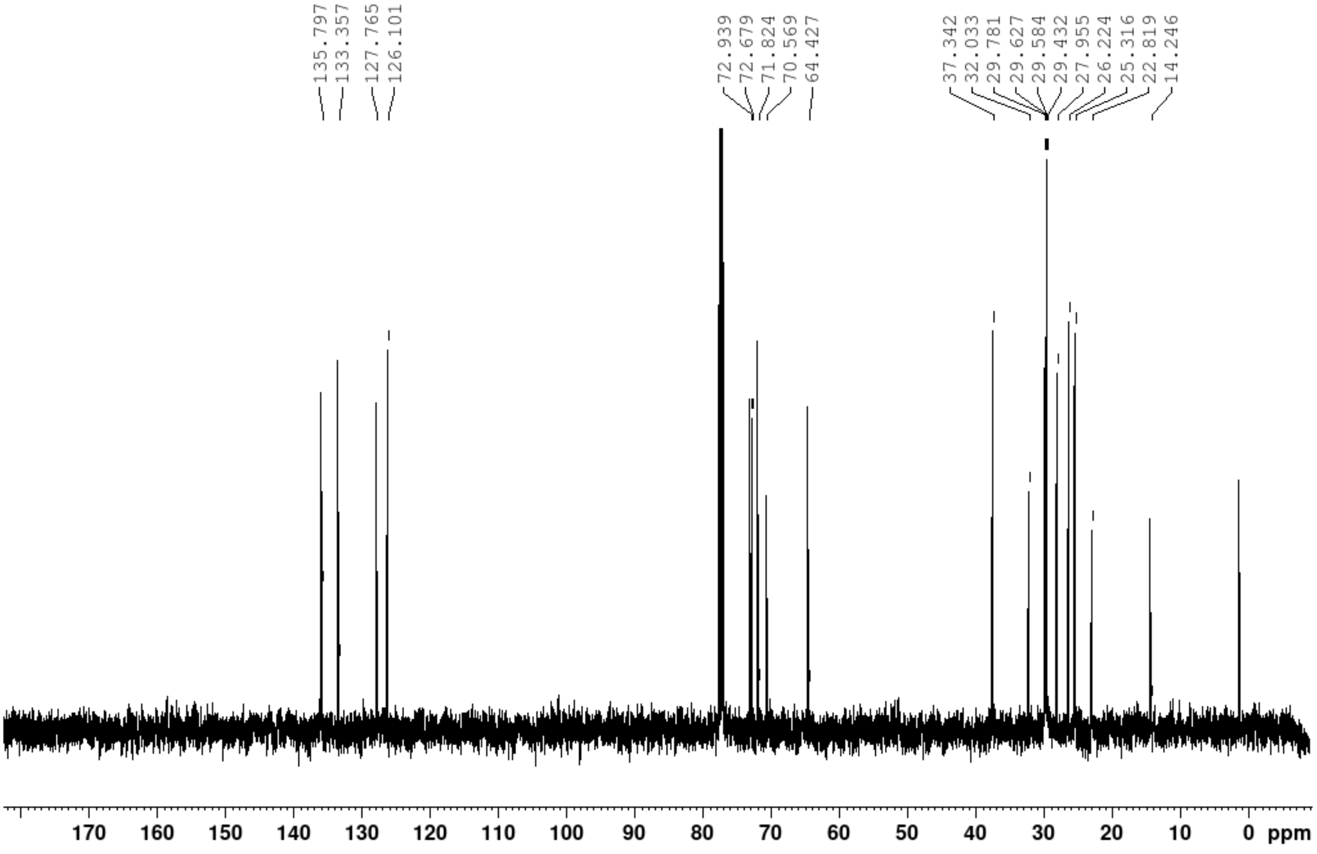

Figure S9: ^{1}H NMR spectrum of synthetic pericharaxin (**1b**) (500 MHz, $CDCl_3$).

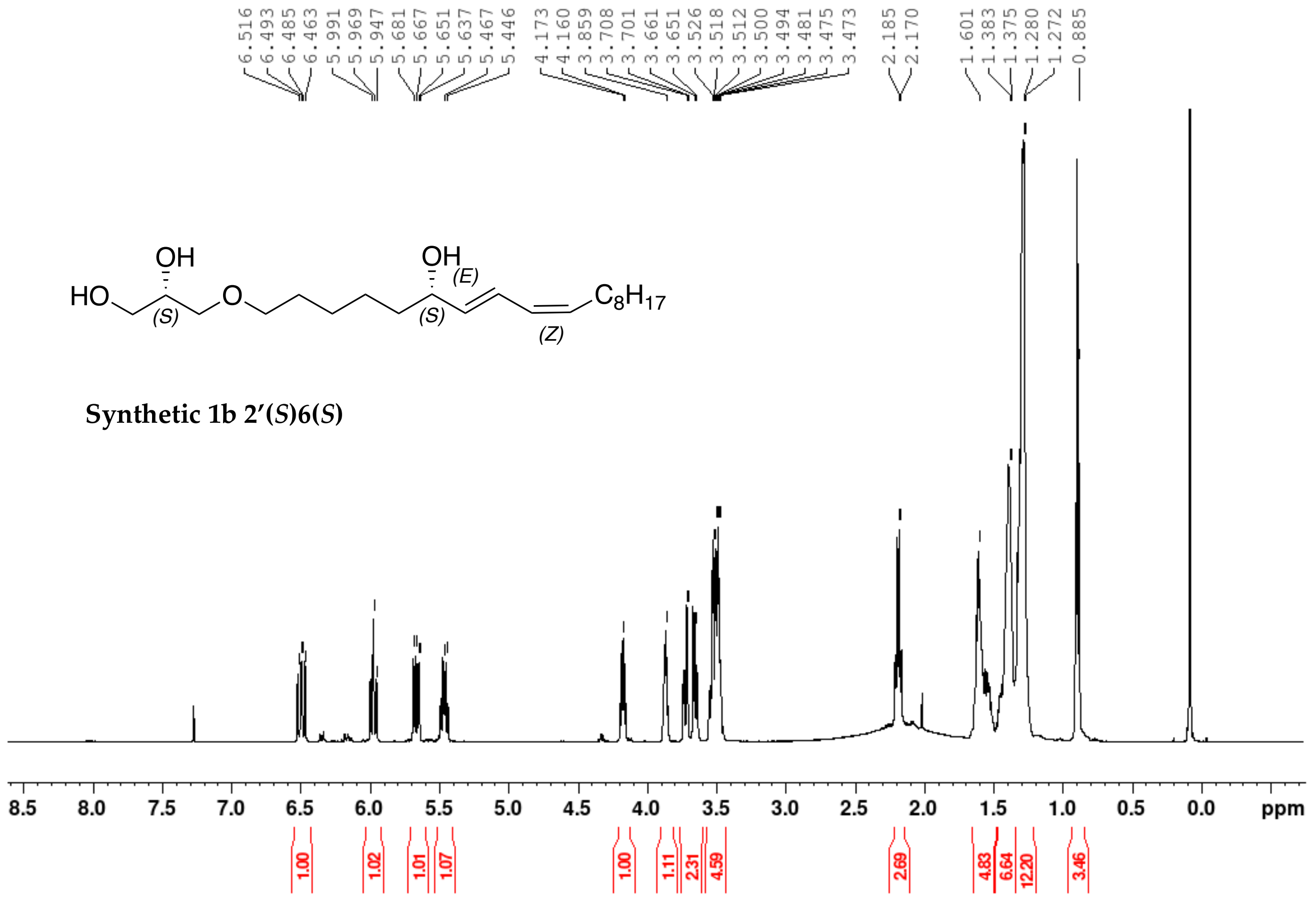

Figure S10: ^{13}C NMR spectrum of synthetic pericharaxin (**1b**) (125 MHz, $CDCl_3$).

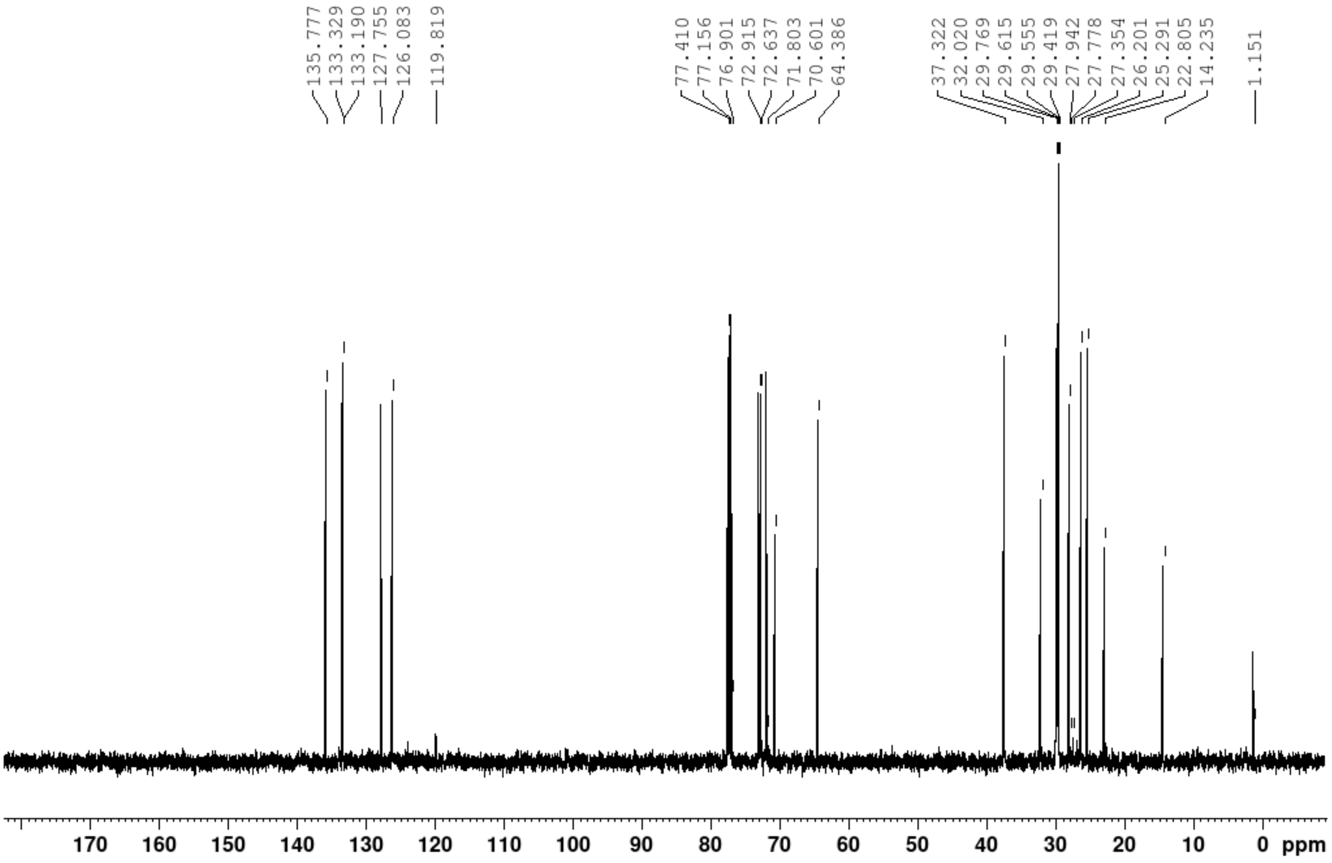

Figure S11: Chiral HPLC chromatograms of natural and synthetic compounds comparison

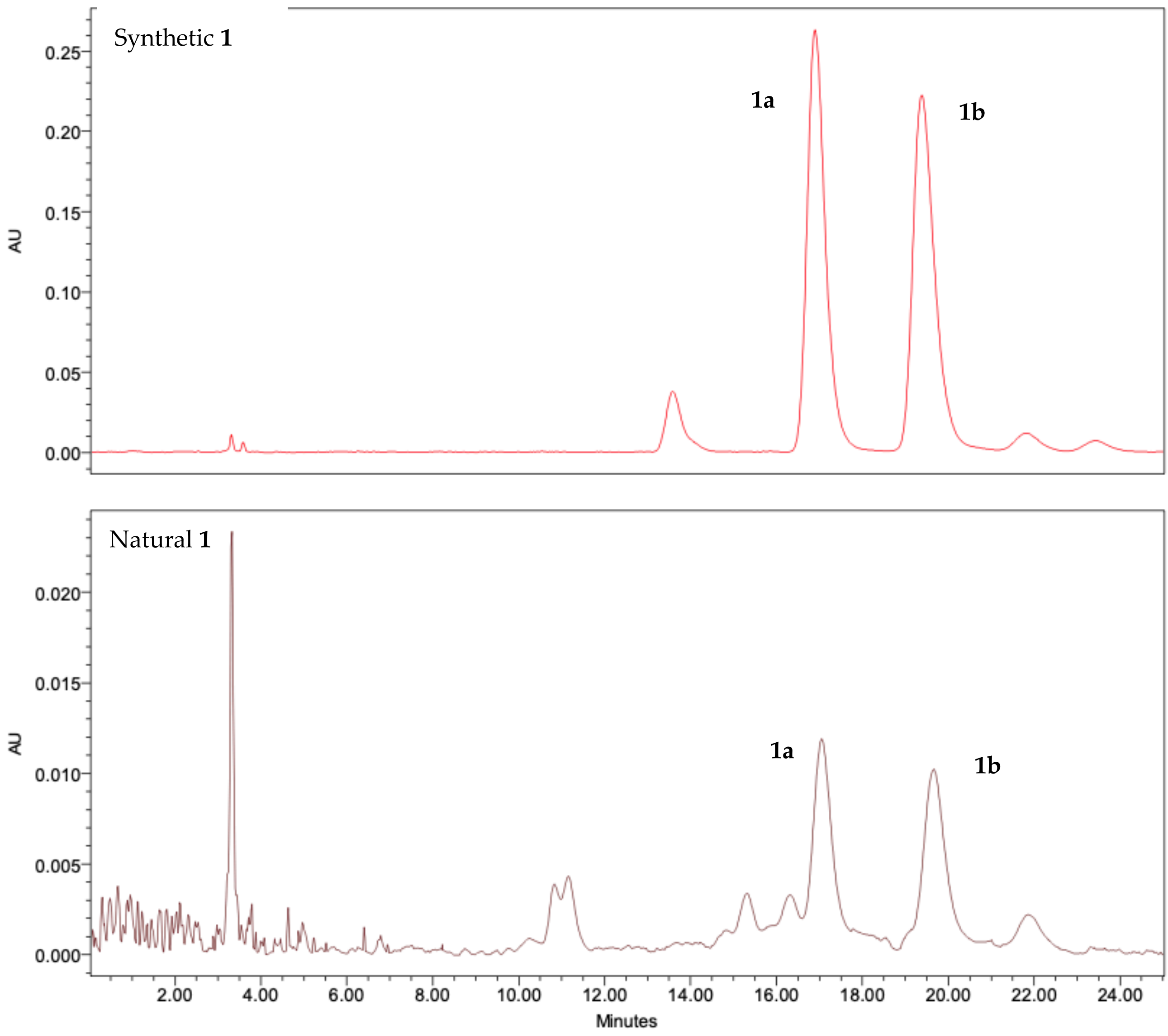

Condition:

Column: CHIRALPAK ID 5μM (4.6x250 mm)

Detection: PDA Max Plot

Solvent: isocratic, heptane/isopropanol 8:2

Figure S12: ^{1}H NMR spectrum of compound **4** (500 MHz, $CDCl_3$).

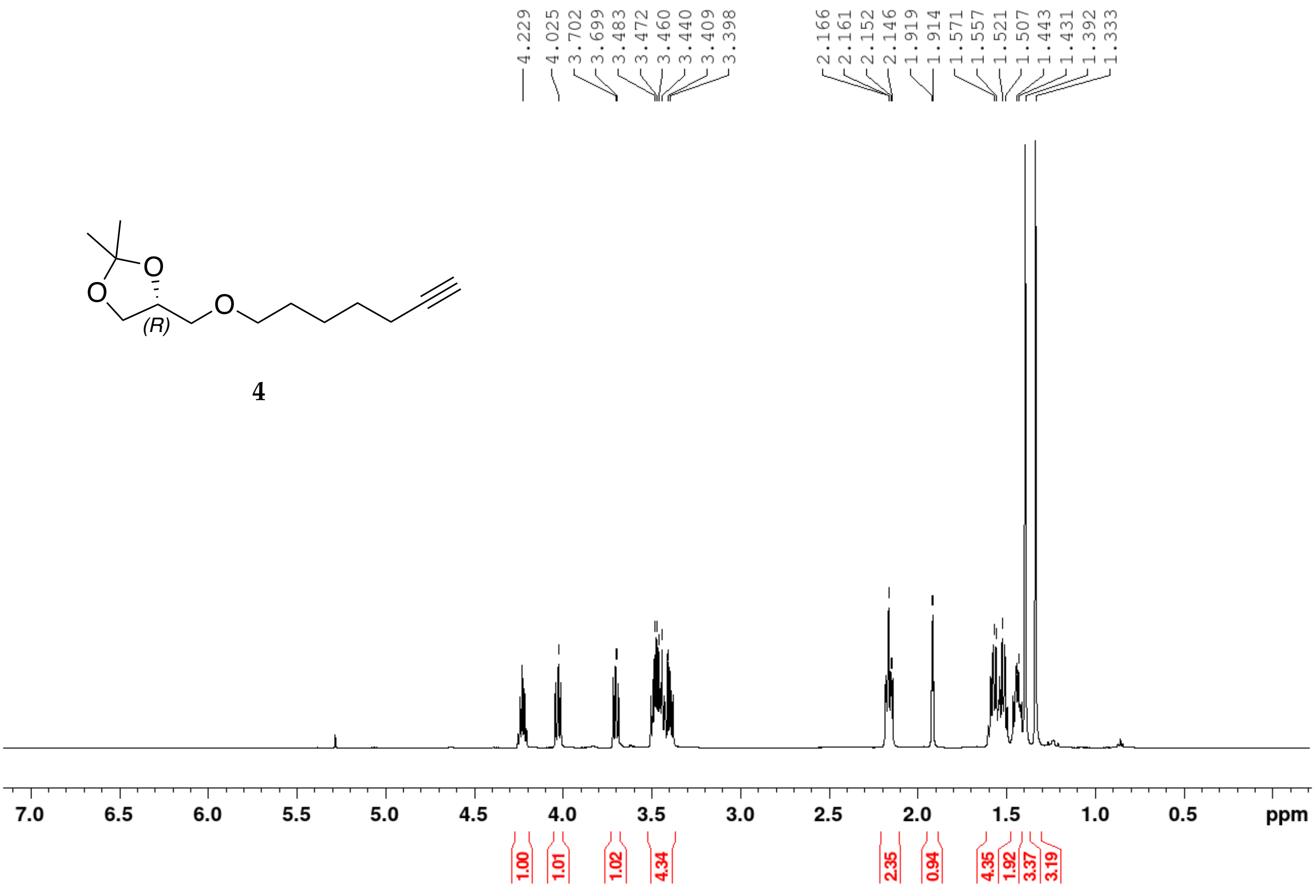

Figure S13: ^{13}C NMR spectrum of compound **4** (125 MHz, $CDCl_3$).

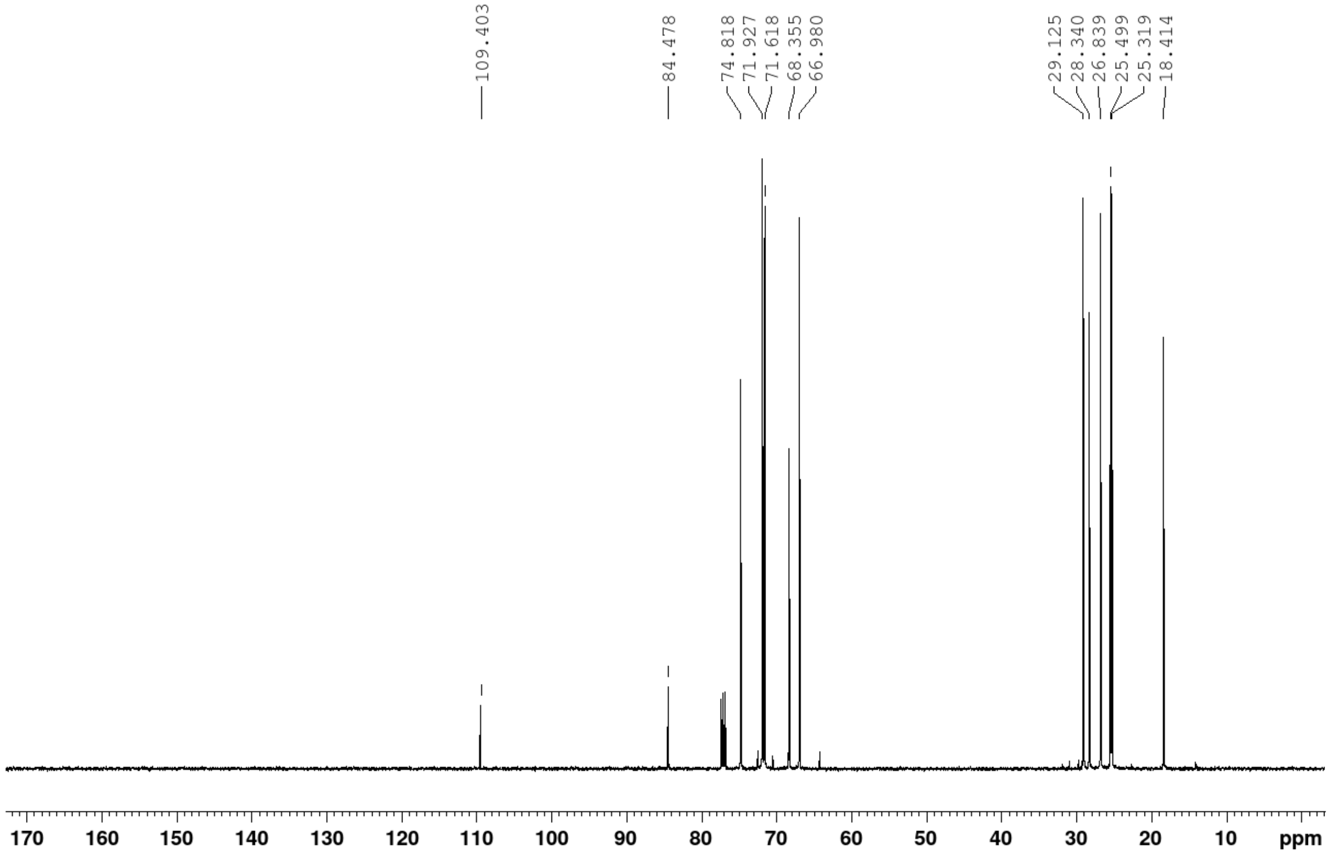

Figure S14: ^{1}H NMR spectrum of compound **5** (500 MHz, $CDCl_3$).

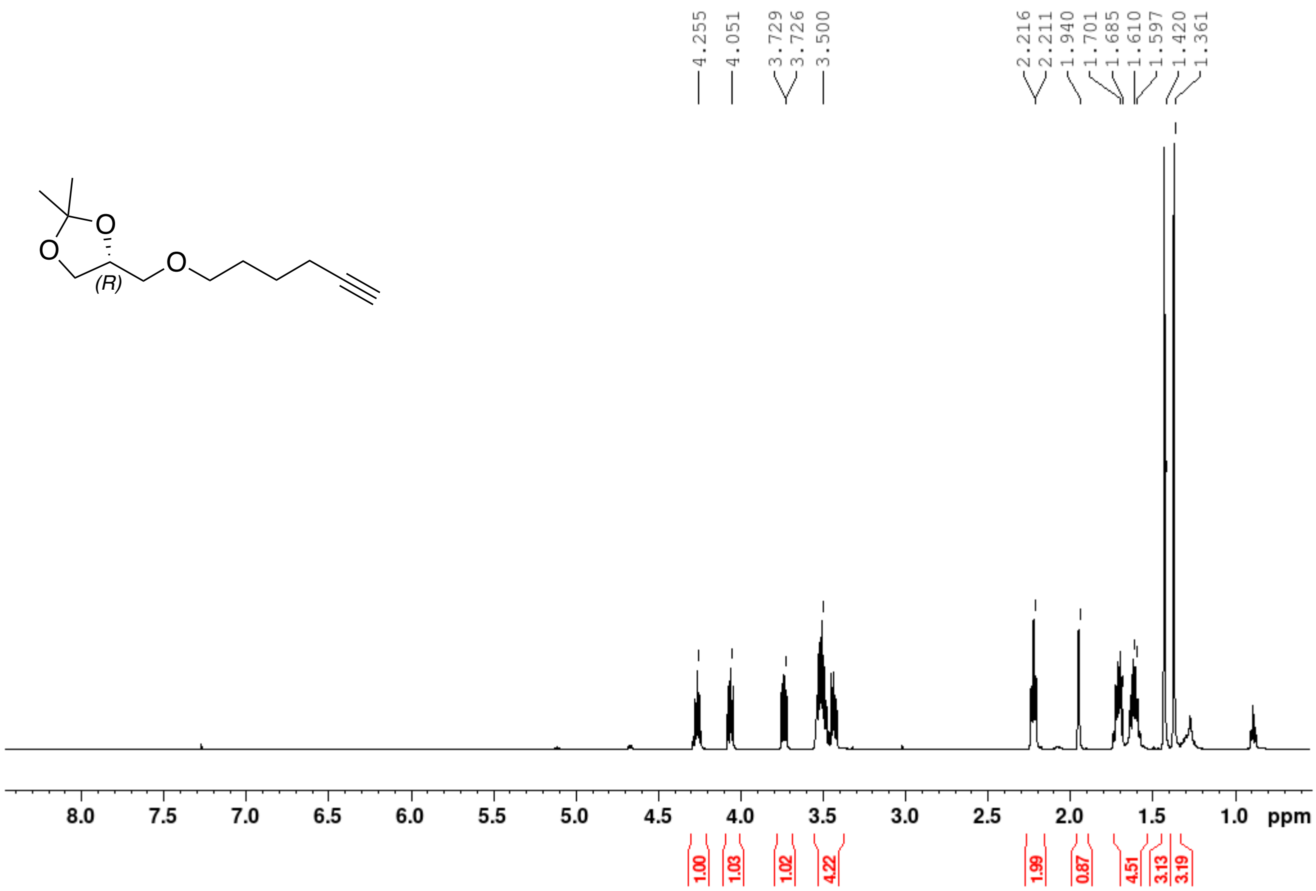

Figure S15: ^{1}H NMR spectrum of compound **6** (500 MHz, $CDCl_3$).

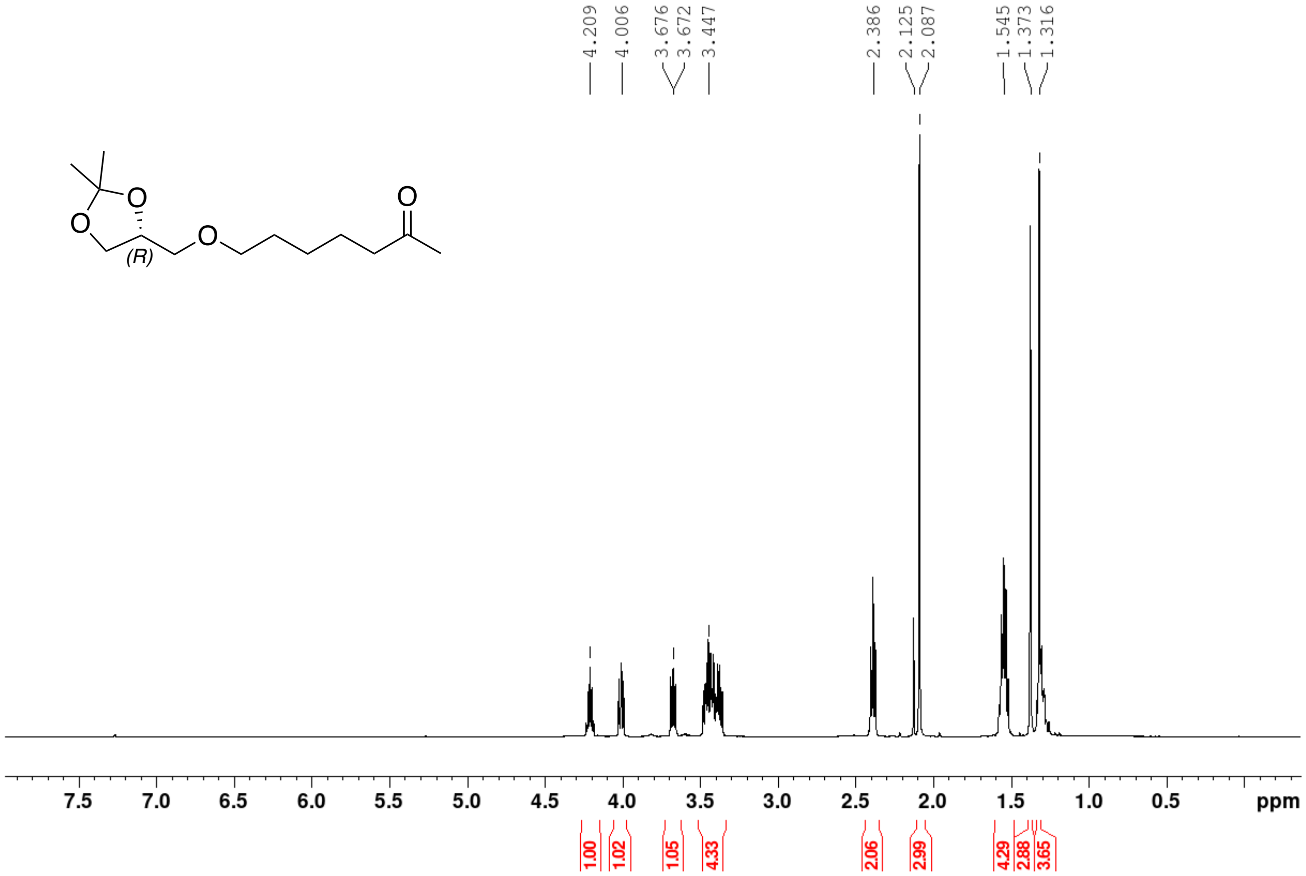

Figure S16: ^{13}C NMR spectrum of compound **6** (125 MHz, $CDCl_3$).

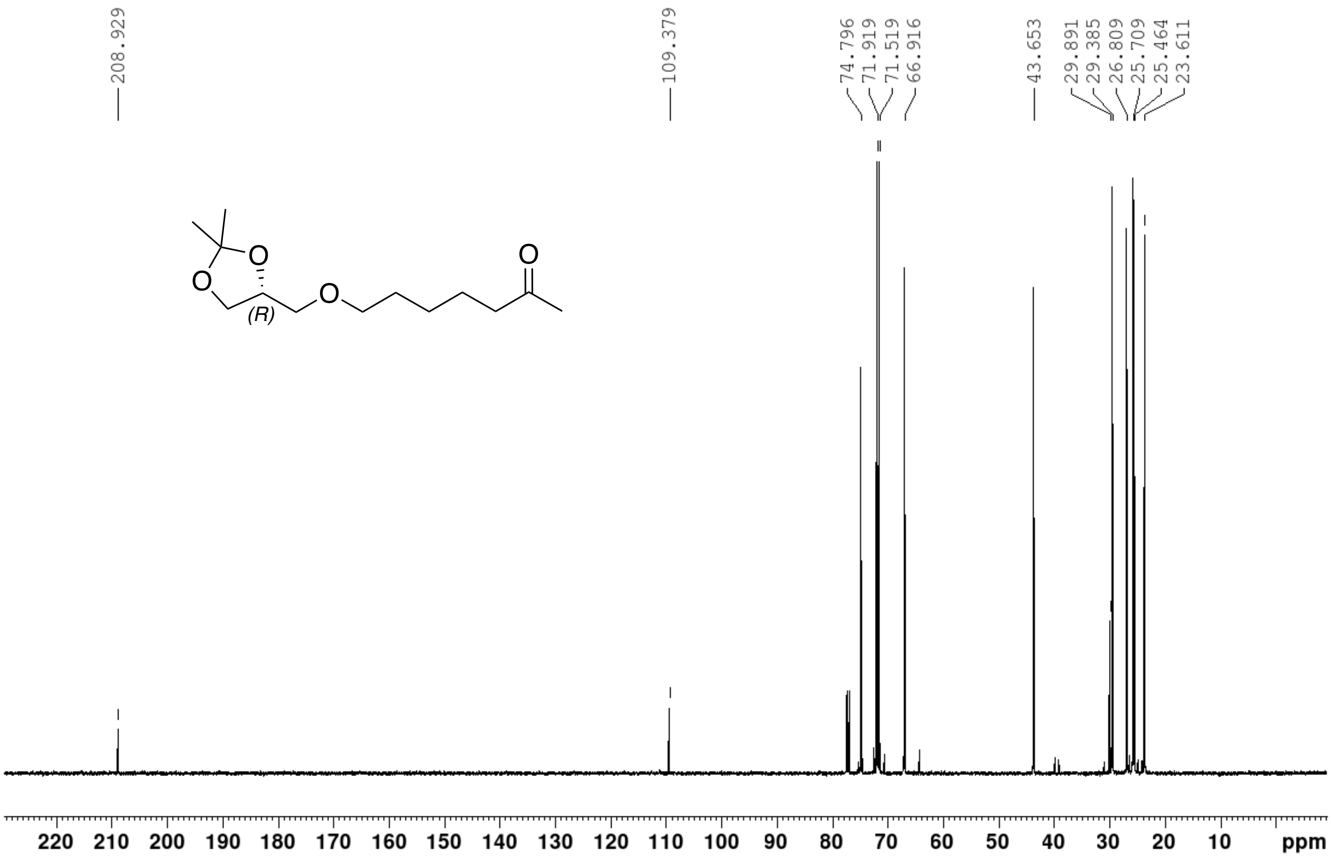

Figure S17: ^{1}H NMR spectrum of compound **7** (500 MHz, CDCl3).

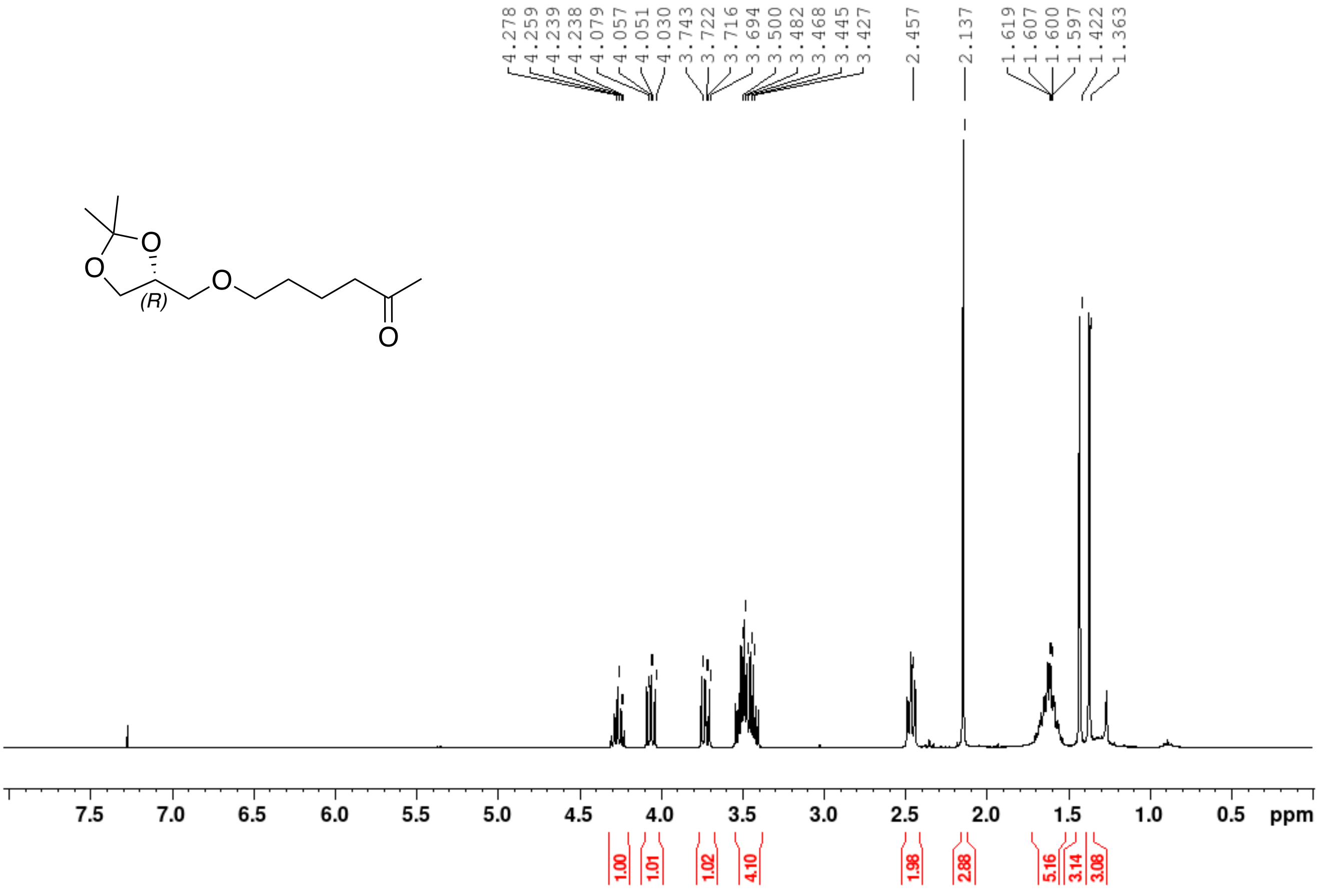

Figure S18: ^{13}C NMR spectrum of compound **7** (125 MHz, $CDCl_3$).

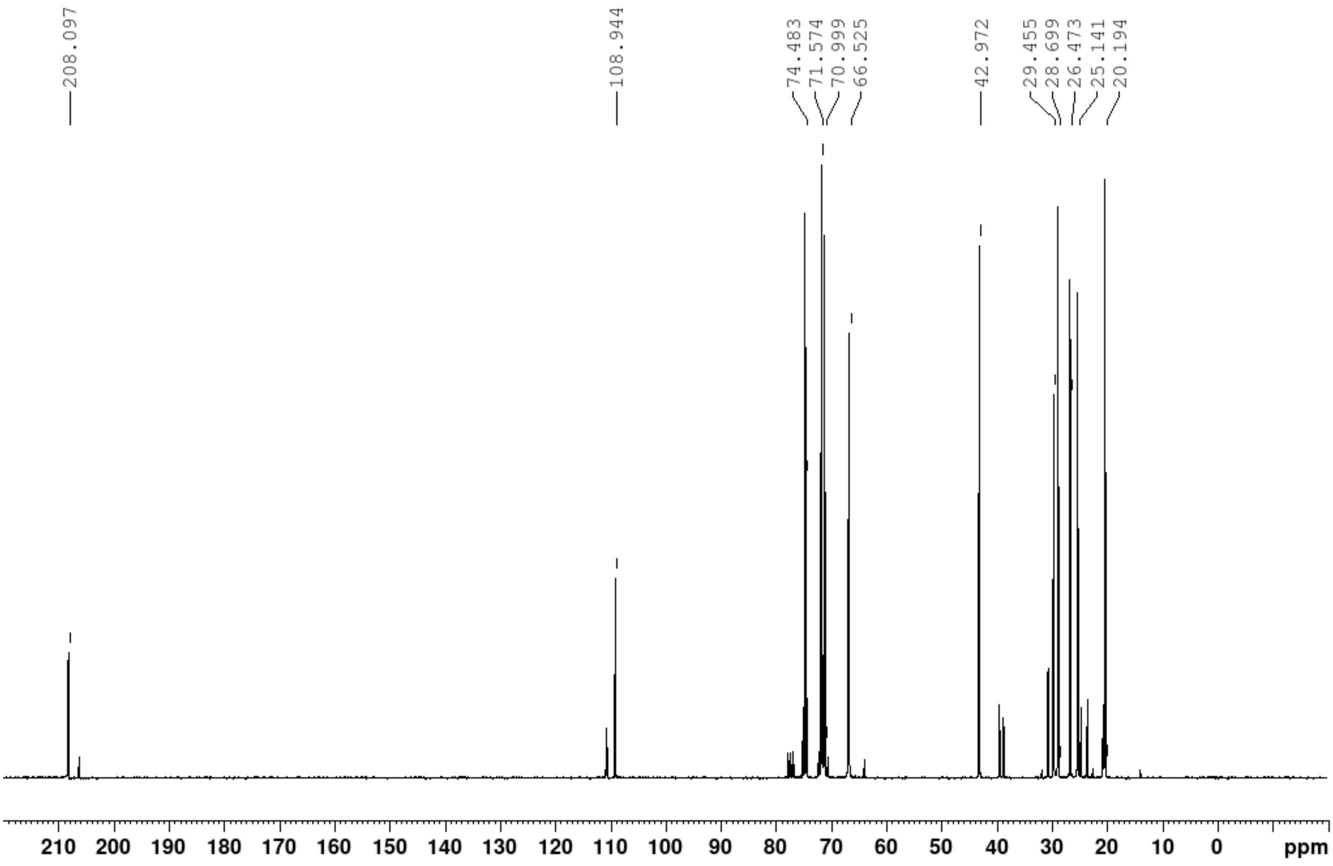

Figure S19: ^{1}H NMR spectrum of compound **11** (300 MHz, $CDCl_3$).

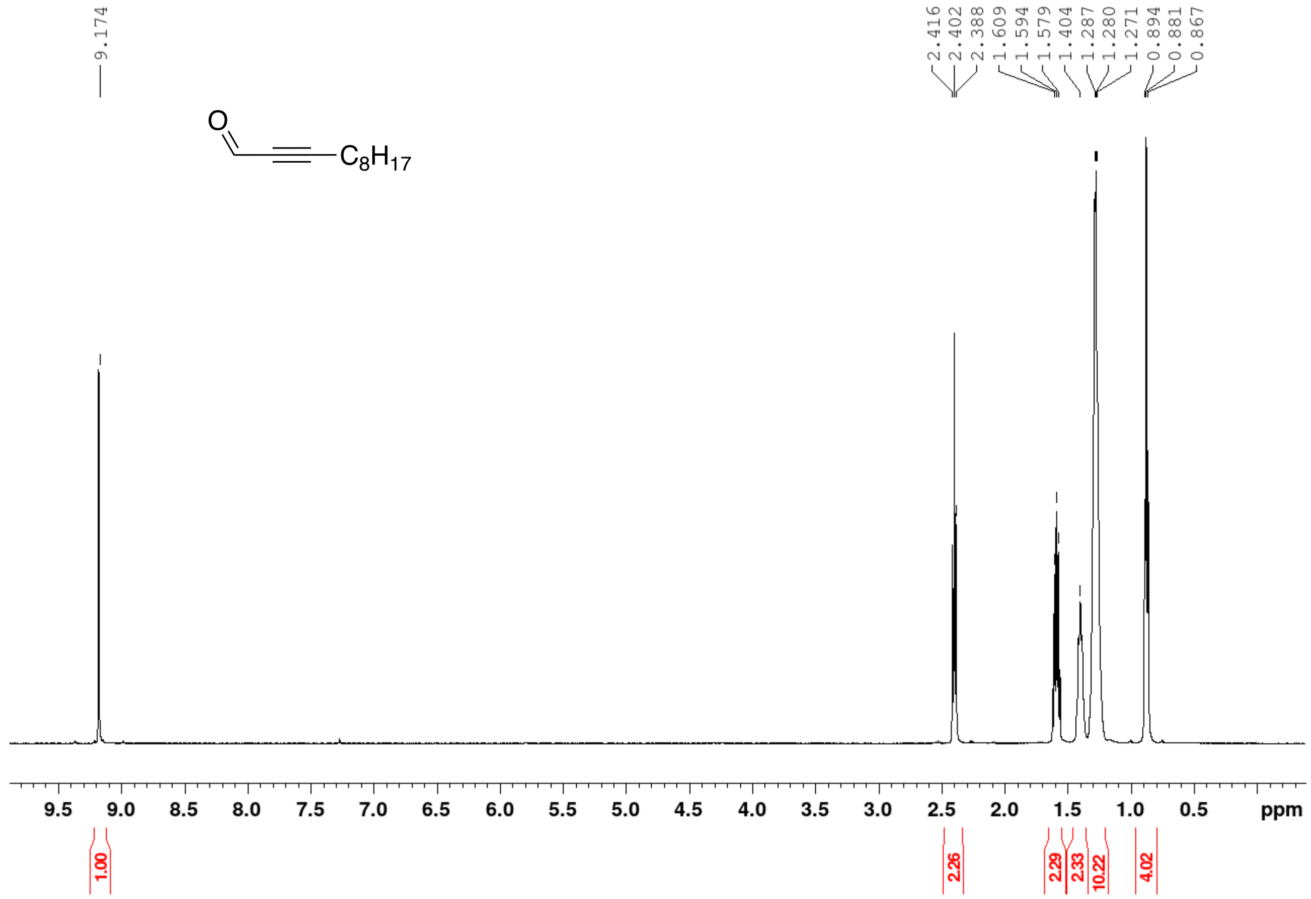

Figure S20: ^{13}C NMR spectrum of compound **11** (75 MHz, $CDCl_3$).

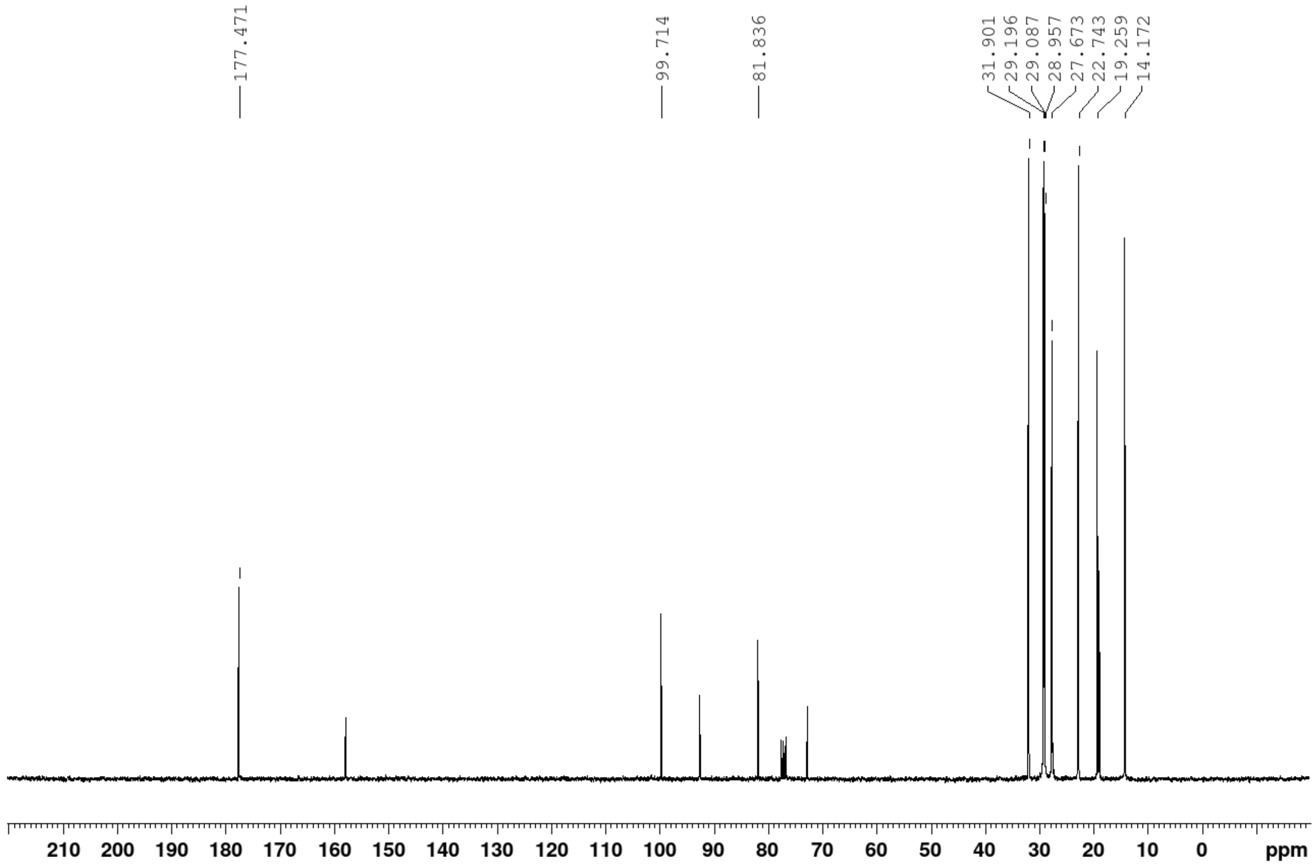

Figure S21: ^{1}H NMR spectrum of compound **12** (500 MHz, $CDCl_3$).

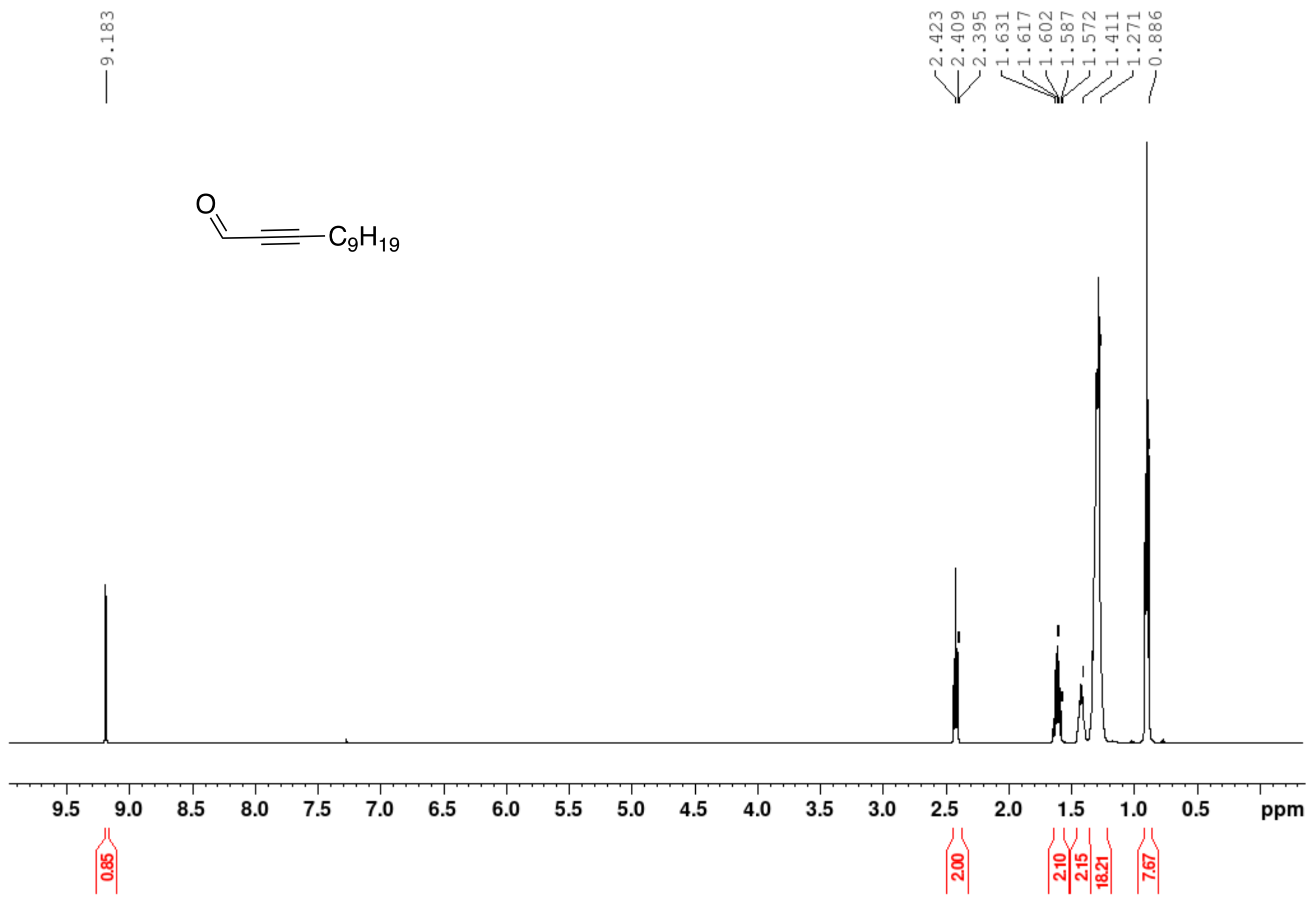

Figure S22: ^{13}C NMR spectrum of compound **12** (125 MHz, $CDCl_3$).

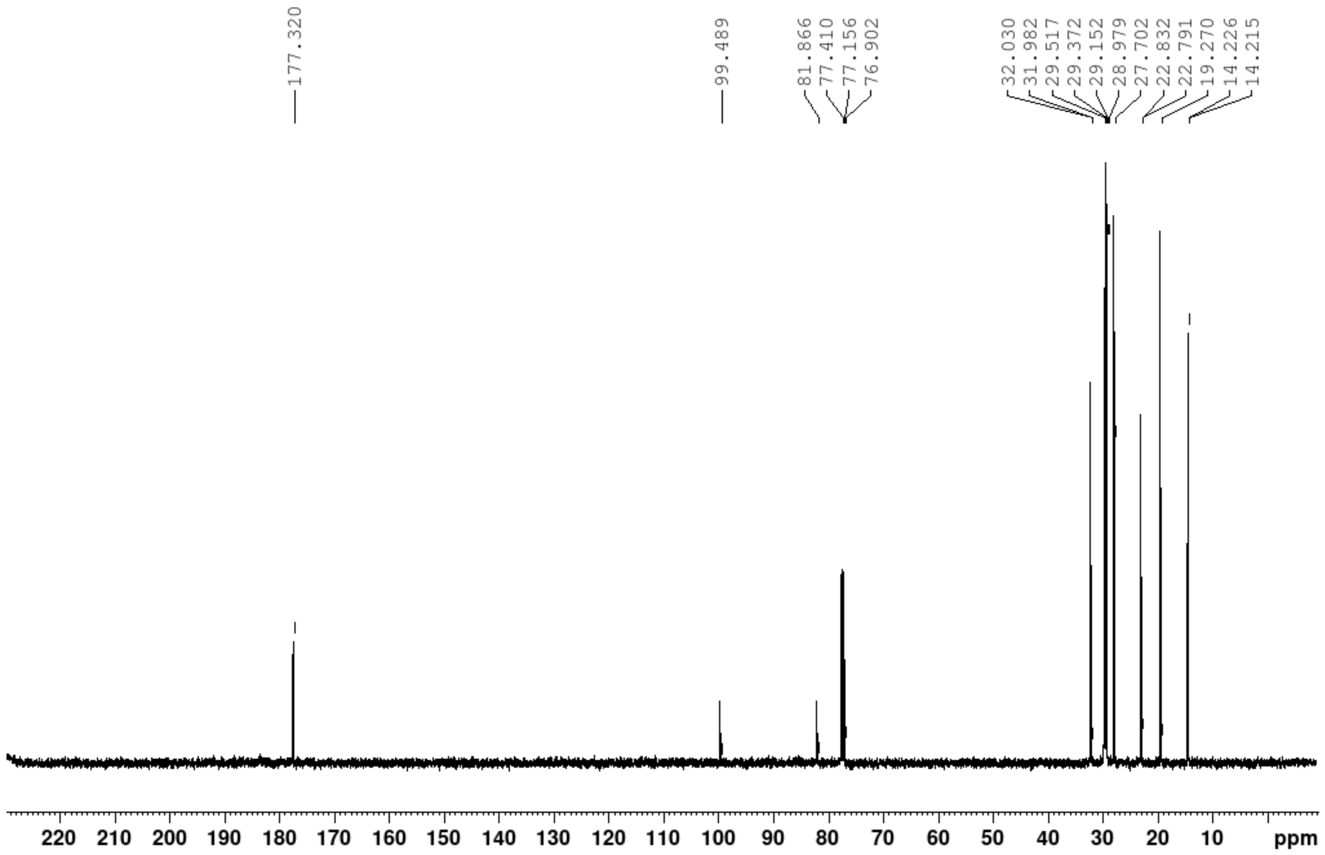

Figure S23: ^{1}H NMR spectrum of compound **13** (300 MHz, $CDCl_3$).

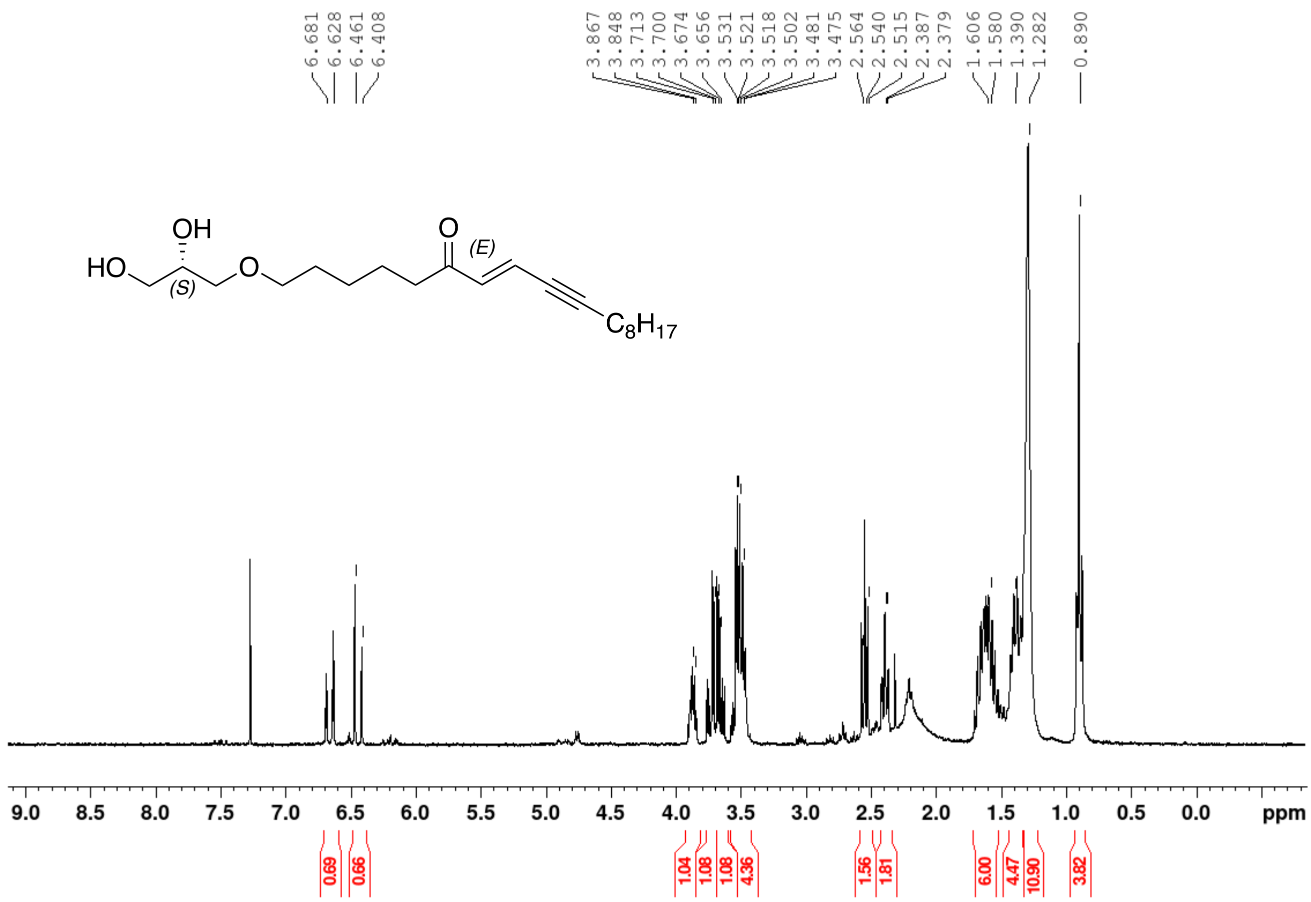

Figure S24: ^{13}C NMR spectrum of compound **13** (75 MHz, $CDCl_3$).

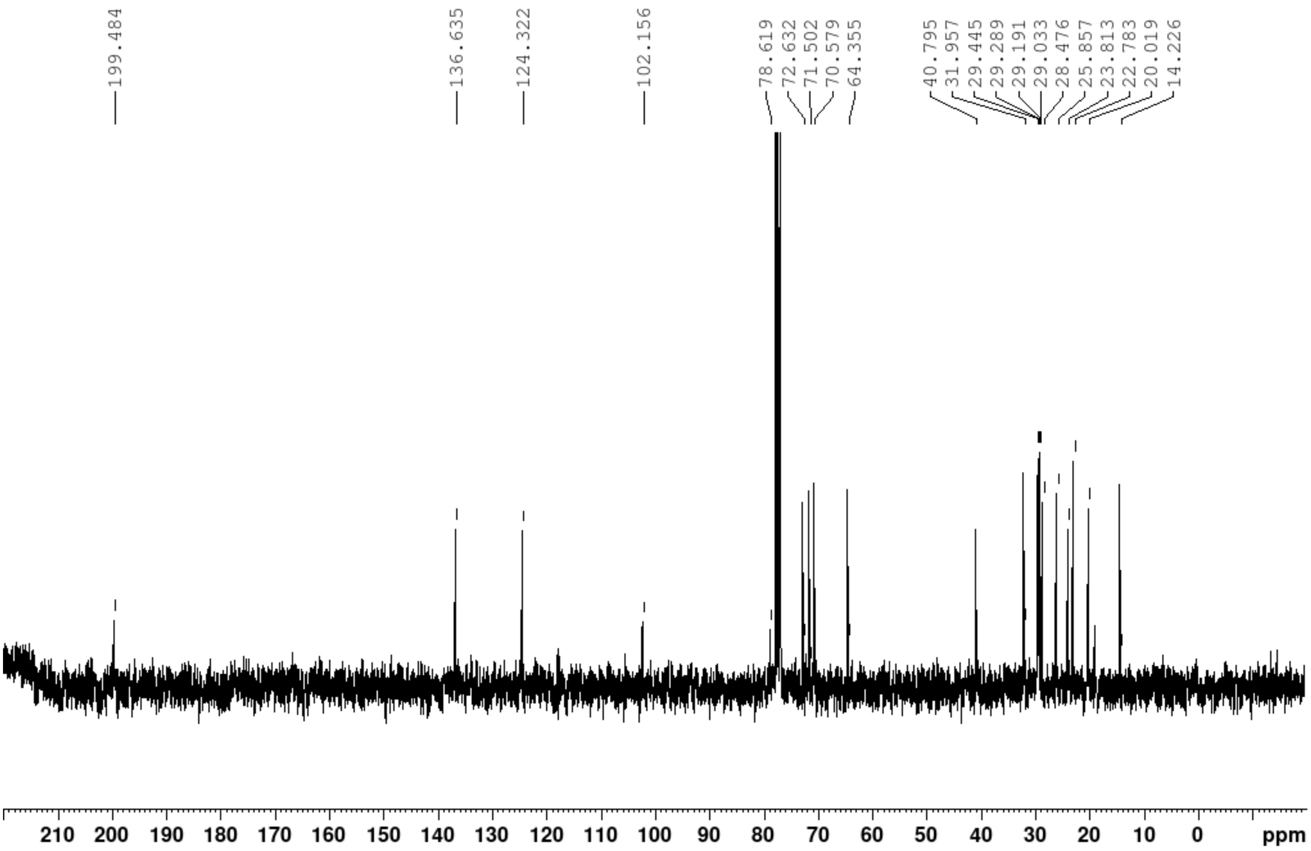

Figure S25: ^{1}H NMR spectrum of compound **14′** (500 MHz, CDCl$_3$).

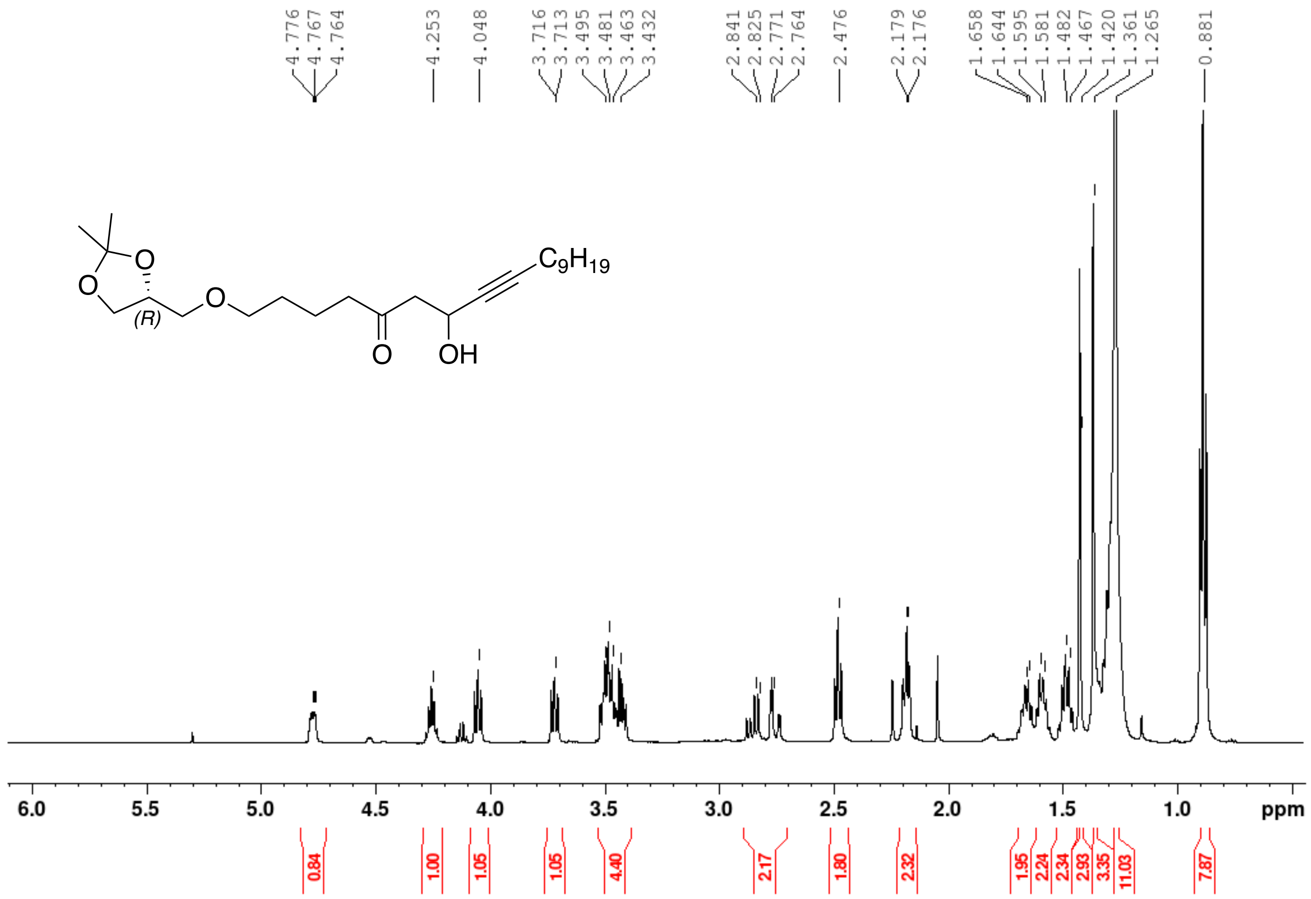

Figure S26: ^{1}H NMR spectrum of compound **14′** (125 MHz, CDCl$_3$).

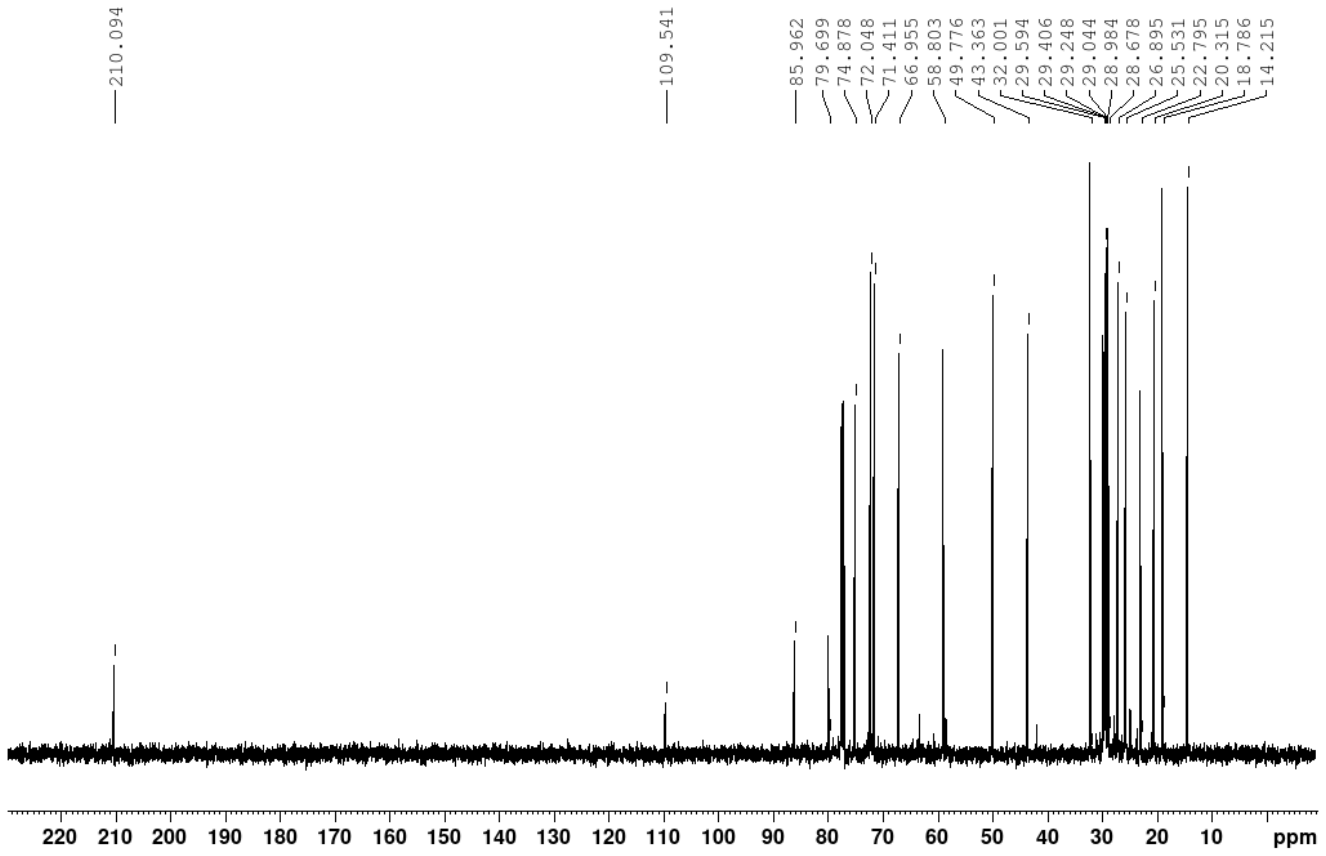

Figure S27: ^{1}H NMR spectrum of compound **14** (500 MHz, $CDCl_3$).

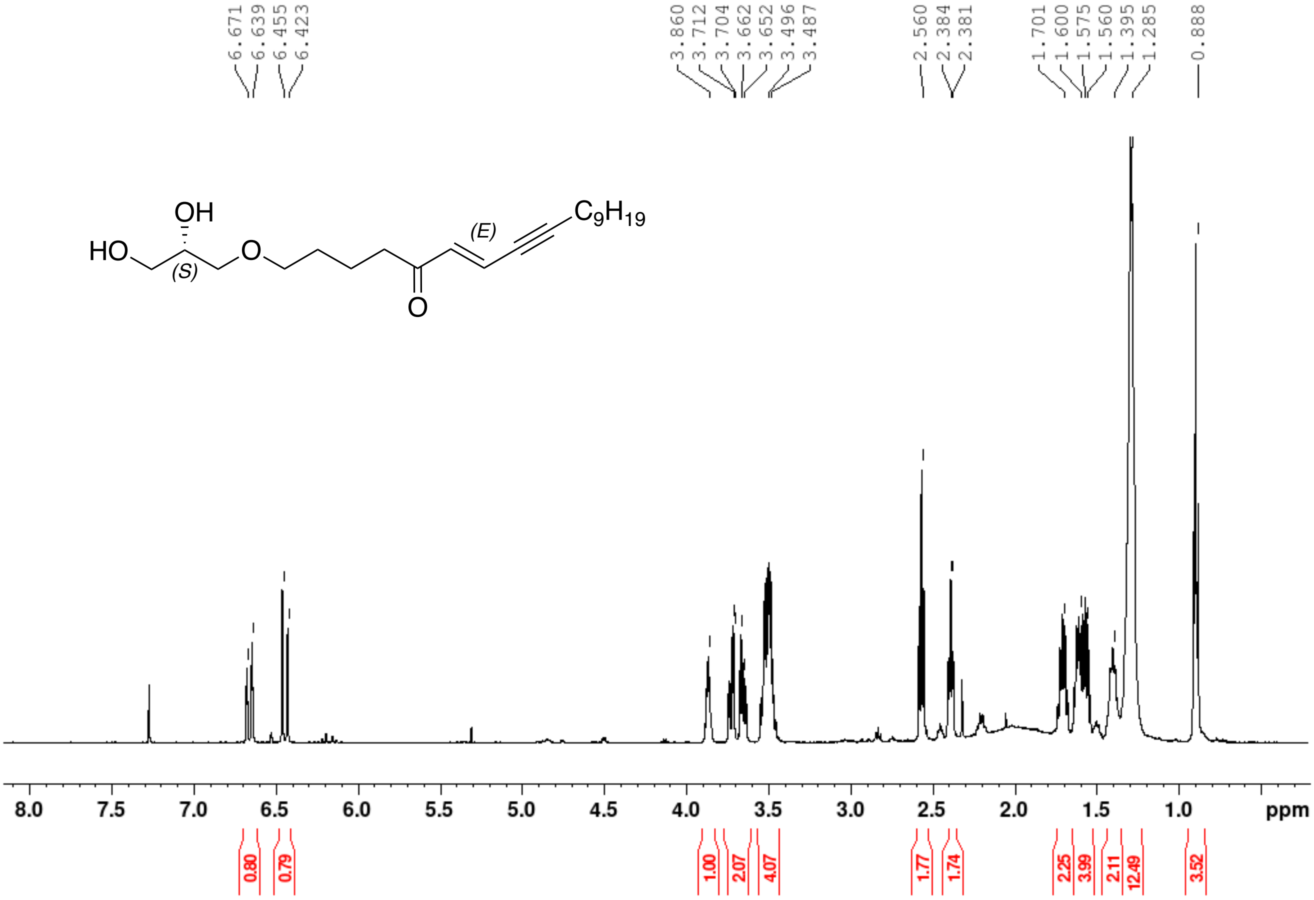

Figure S28: ^{13}C NMR spectrum of compound **14** (125 MHz, $CDCl_3$).

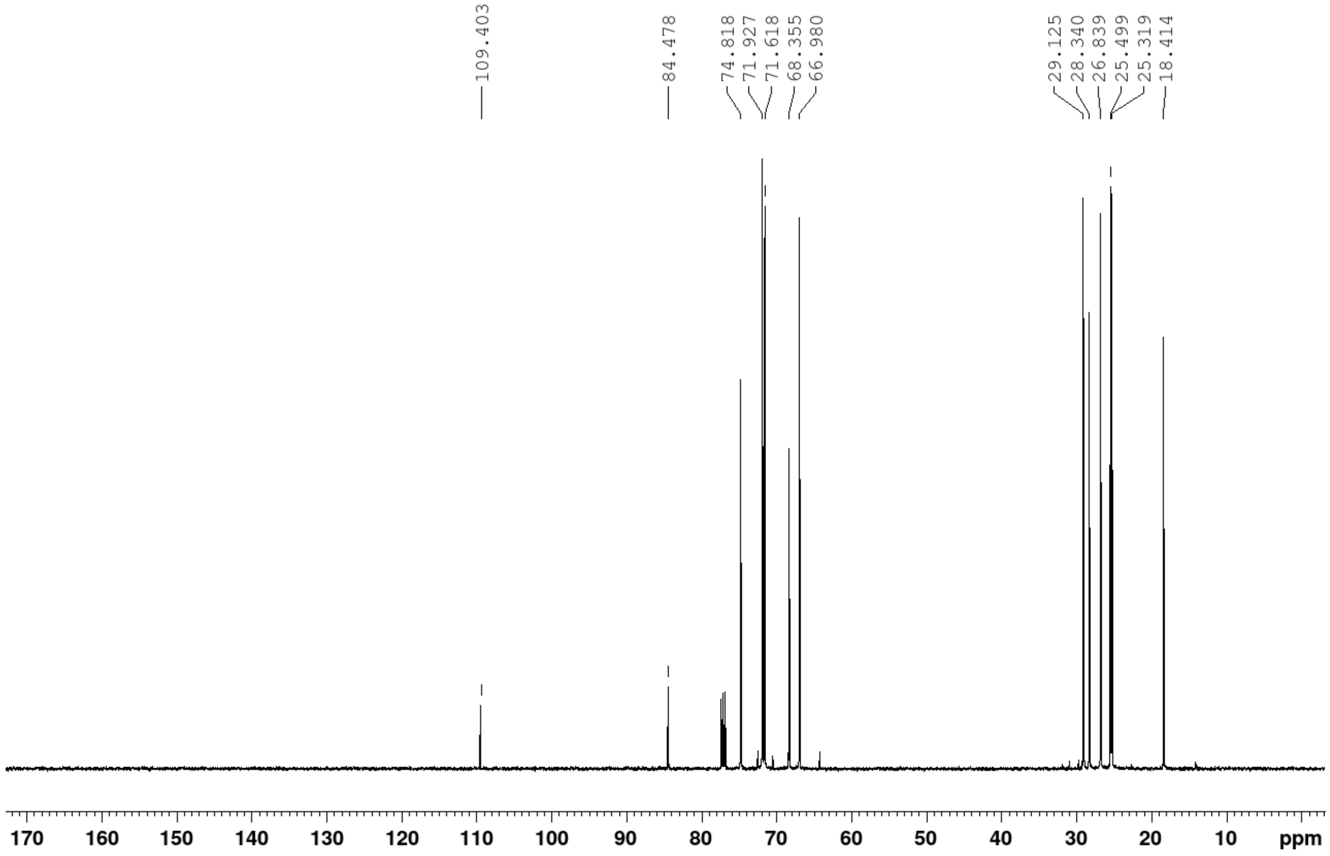

Figure S29: NMR comparisons of natural product with the two synthetic products

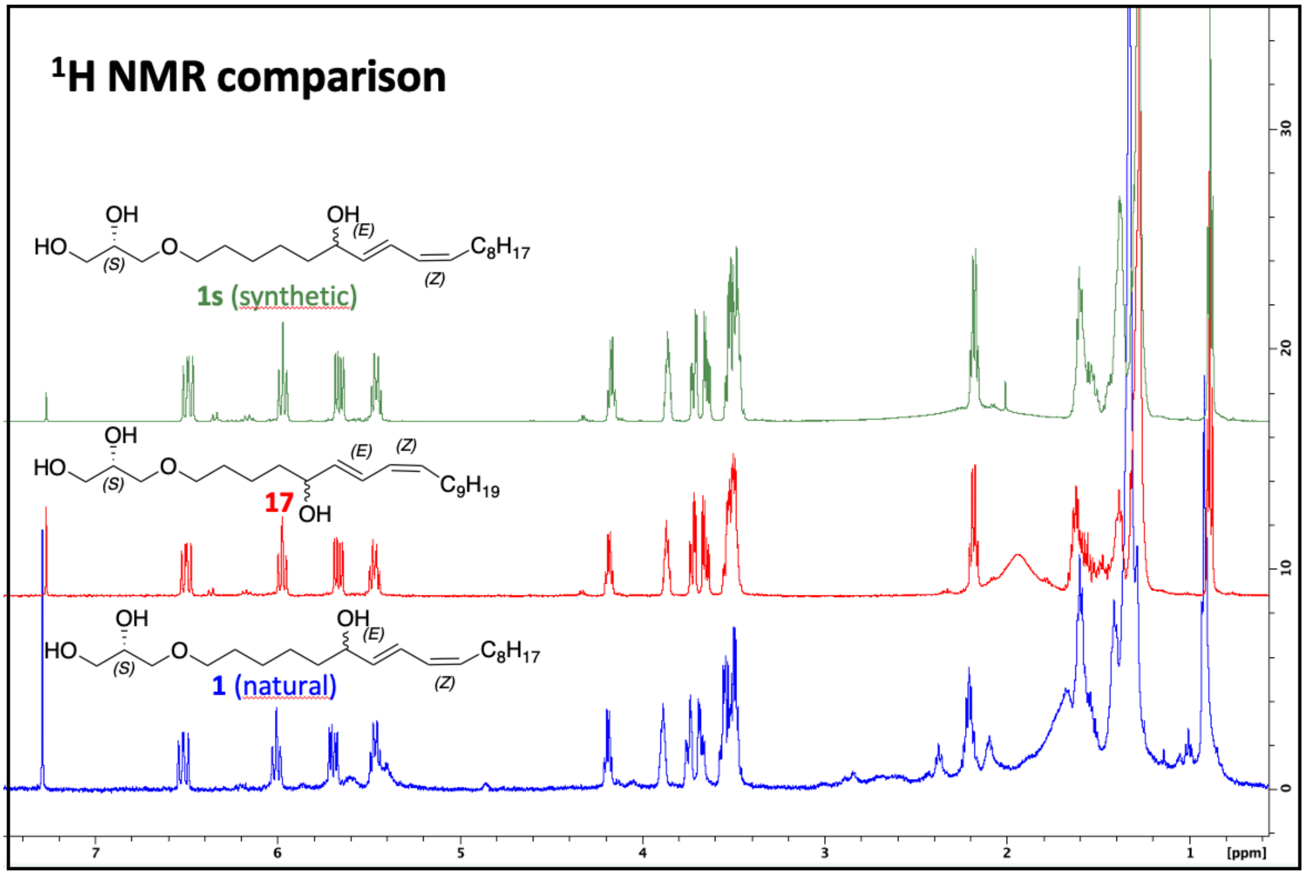

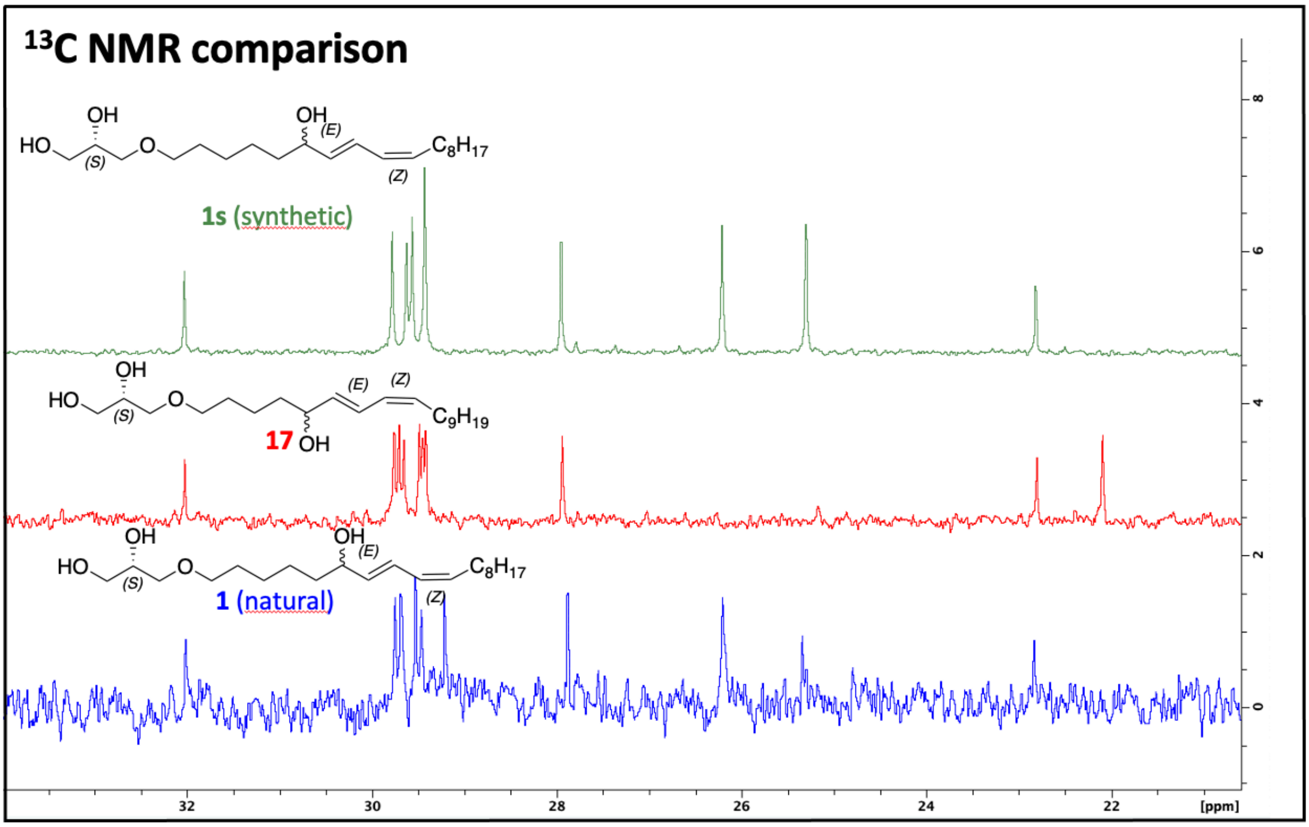

Figure S30: ^{13}C NMR comparison of epimers **1a** (*S*,*R*) and **1b** (*S*,*S*)

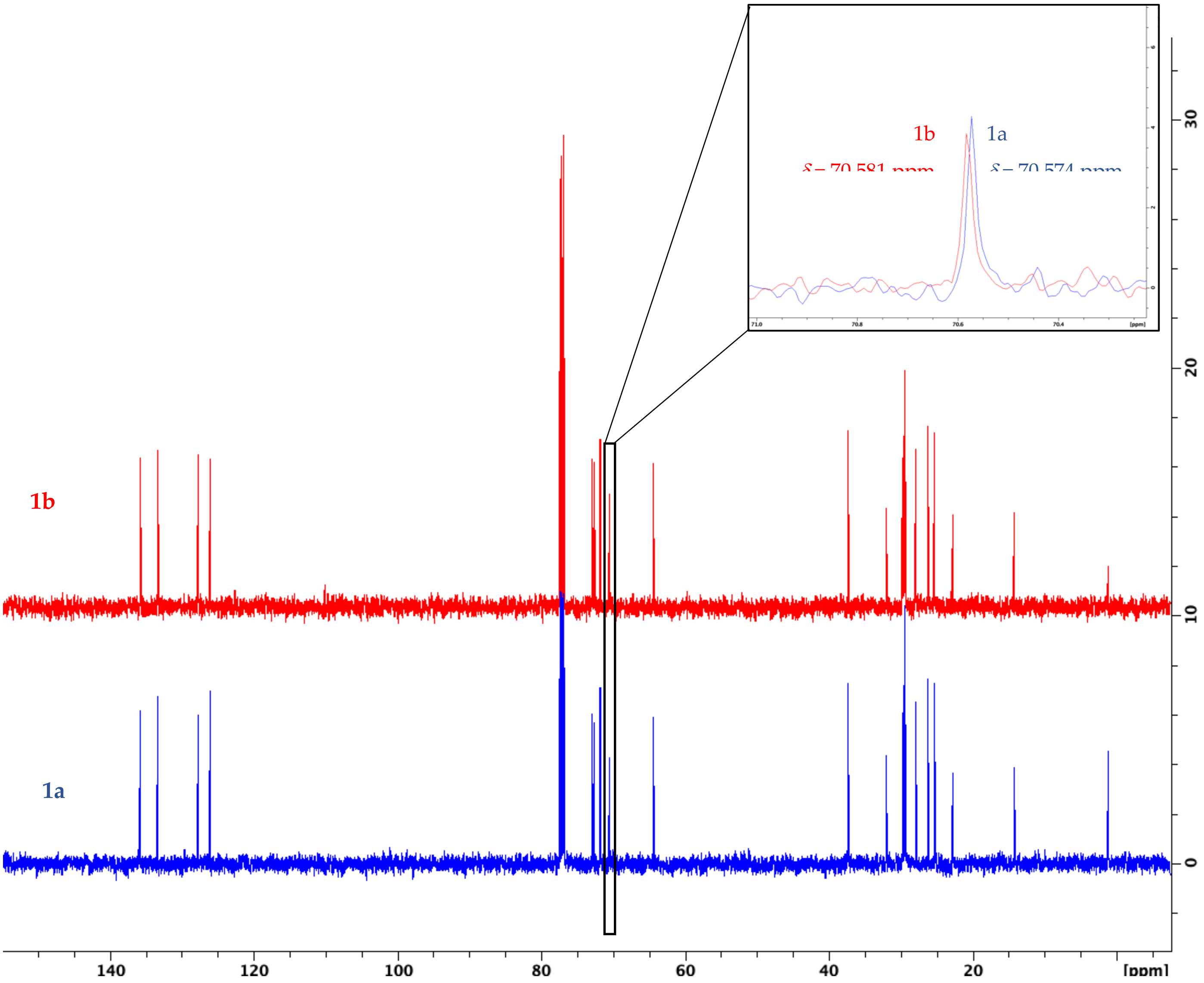

Figure S31: results of the assessment of an ATDC5-based rapid screening platform using the human Col X (hCol X) proximal promoter activity for selection of natural compounds able to stimulate endochondral differentiation. hColX-promoter activity in murine ATDC5 chondrocyte line cultured under endochondral differentiation conditions as assessed by measurement of luminometric luciferase signals.

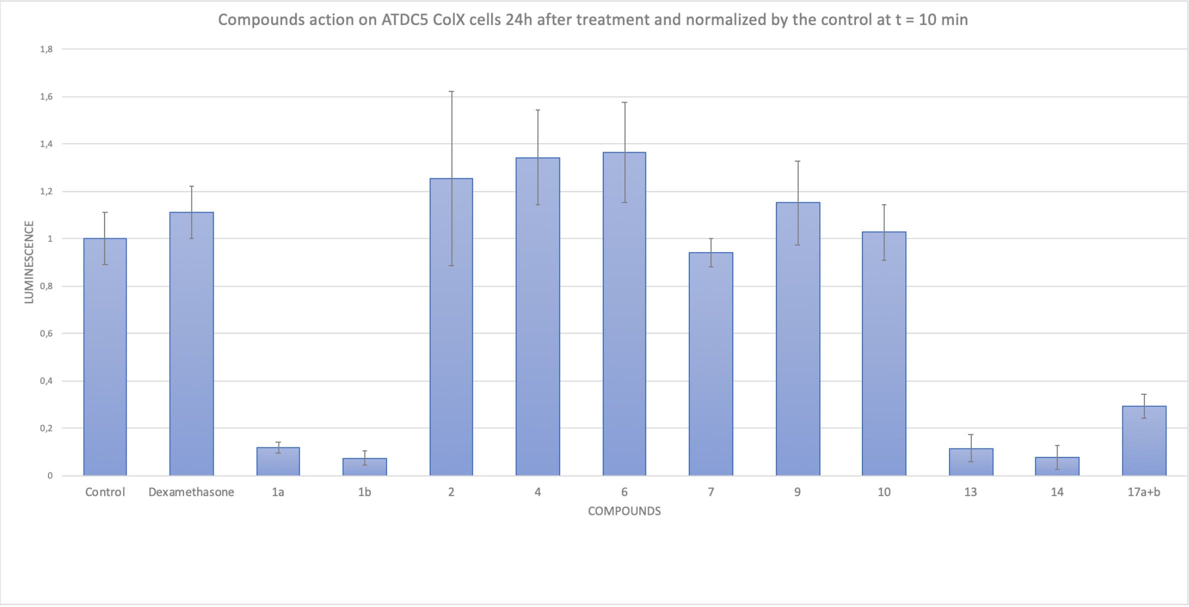

Supplement: Supplementary file 1 [file marinedrugs-20-00635-s001.zip › marinedrugs-1957367-supplementary.pdf]
